# Differential mitochondrial roles for α-synuclein in DRP1-dependent fission and PINK1/Parkin-mediated oxidation

**DOI:** 10.1038/s41419-021-04046-3

**Published:** 2021-08-17

**Authors:** Thomas J. Krzystek, Rupkatha Banerjee, Layne Thurston, JianQiao Huang, Kelsey Swinter, Saad Navid Rahman, Tomas L. Falzone, Shermali Gunawardena

**Affiliations:** 1 Department of Biological Sciences, The State University of New York at Buffalo, Buffalo, NY 14260 USA; 2 Instituto de Biología Celular y Neurociencias IBCN (CONICET-UBA), Universidad De Buenos Aires, Buenos Aires, Argentina; 3 Instituto de Investigación en Biomedicina de Buenos Aires (IBioBA), Partner Institute of the Max Planck Society, Buenos Aires, Argentina

**Keywords:** Mechanisms of disease, Parkinson's disease

## Abstract

Mitochondria are highly dynamic organelles with strict quality control processes that maintain cellular homeostasis. Within axons, coordinated cycles of fission-fusion mediated by dynamin related GTPase protein (DRP1) and mitofusins (MFN), together with regulated motility of healthy mitochondria anterogradely and damaged/oxidized mitochondria retrogradely, control mitochondrial shape, distribution and size. Disruption of this tight regulation has been linked to aberrant oxidative stress and mitochondrial dysfunction causing mitochondrial disease and neurodegeneration. Although pharmacological induction of Parkinson’s disease (PD) in humans/animals with toxins or in mice overexpressing α-synuclein (α-syn) exhibited mitochondrial dysfunction and oxidative stress, mice lacking α-syn showed resistance to mitochondrial toxins; yet, how α-syn influences mitochondrial dynamics and turnover is unclear. Here, we isolate the mechanistic role of α-syn in mitochondrial homeostasis in vivo in a humanized *Drosophila* model of Parkinson’s disease (PD). We show that excess α-syn causes fragmented mitochondria, which persists with either truncation of the C-terminus (α-syn^1–120^) or deletion of the NAC region (α-syn^ΔNAC^). Using in vivo oxidation reporters Mito-roGFP2-ORP1/GRX1 and MitoTimer, we found that α-syn-mediated fragments were oxidized/damaged, but α-syn^1–120^-induced fragments were healthy, suggesting that the C-terminus is required for oxidation. α-syn-mediated oxidized fragments showed biased retrograde motility, but α-syn^1–120^-mediated healthy fragments did not, demonstrating that the C-terminus likely mediates the retrograde motility of oxidized mitochondria. Depletion/inhibition or excess DRP1-rescued α-syn-mediated fragmentation, oxidation, and the biased retrograde motility, indicating that DRP1-mediated fragmentation is likely upstream of oxidation and motility changes. Further, excess PINK/Parkin, two PD-associated proteins that function to coordinate mitochondrial turnover via induction of selective mitophagy, rescued α-syn-mediated membrane depolarization, oxidation and cell death in a C-terminus-dependent manner, suggesting a functional interaction between α-syn and PINK/Parkin. Taken together, our findings identify distinct roles for α-syn in mitochondrial homeostasis, highlighting a previously unknown pathogenic pathway for the initiation of PD.

## Introduction

α-syn is a soluble, natively unfolded cytosolic protein that becomes structured when bound to phospholipids [1, 2]. Although α-syn lacks a true mitochondrial localization sequence, NMR studies suggest that α-syn contains a cryptic mitochondrial targeting sequence that can facilitate anchoring α-syn to mitochondrial membranes [2, 3]. Indeed, studies have shown that α-syn can localize to the intermembrane space, to the matrix [3–6] or to mitochondria-associated ER membranes [7]. Further, excess α-syn caused mitochondrial fragmentation in *C. elegans* [8, 9], in dorsal root ganglia of zebrafish [10], and in mammalian neuronal cell lines [4, 6, 9]. Reductions in ATP levels, membrane potential and complex I deficits have also been correlated with α-syn aggregation in both cultured cells and transgenic mice [11, 12]. However, there is no consensus as to how α-syn effects mitochondrial homeostasis: whether biophysical properties of α-syn associations with mitochondrial membranes and/or whether functional interactions with mitochondrial shaping/turnover proteins dictate α-syn-mediated mitochondrial defects.

Here, we use a humanized *Drosophila* α-syn-mediated PD model, *Drosophila* genetics, and pharmacological agents together with in vivo mitochondrial quality control reporters, to isolate how α-syn effects mitochondrial homeostasis at the single mitochondrial level, within a whole organism. We show that excess α-syn cause fragmented and oxidized mitochondria independent of α-syn aggregation. Since deletion of the NAC region or the C-terminal region did not eliminate mitochondrial fragmentation, we propose that the N-terminus of α-syn likely plays a role in mitochondrial fragmentation via a DRP1-dependent mechanism. Since deletion of the C-terminal region rescued mitochondrial oxidation and the biased retrograde motility, we postulate that the C-terminus of α-syn induces mitochondrial oxidation via a PINK1/Parkin-mediated pathway and enables the retrograde motility of damaged mitochondria. Together our results propose that the structural properties of distinct regions of α-syn likely exert diverse biophysical interactions with mitochondria. Therefore, continuous or transient associations between α-syn and mitochondrial fission/fusion proteins, mitochondrial turnover proteins, oxidative factors and molecular motors likely contribute to mitochondrial dysfunction seen in PD. Our findings demonstrate a novel physiological mechanism for α-syn in mitochondrial homeostasis, and highlight a common pathogenic pathway that can be targeted for therapeutics early before neuronal loss or clinical manifestation of PD and/or other synucleinopathies.

## Results

### Excess α-syn causes mitochondrial fragmentation independent of α-syn aggregation

**Fig. 1:**
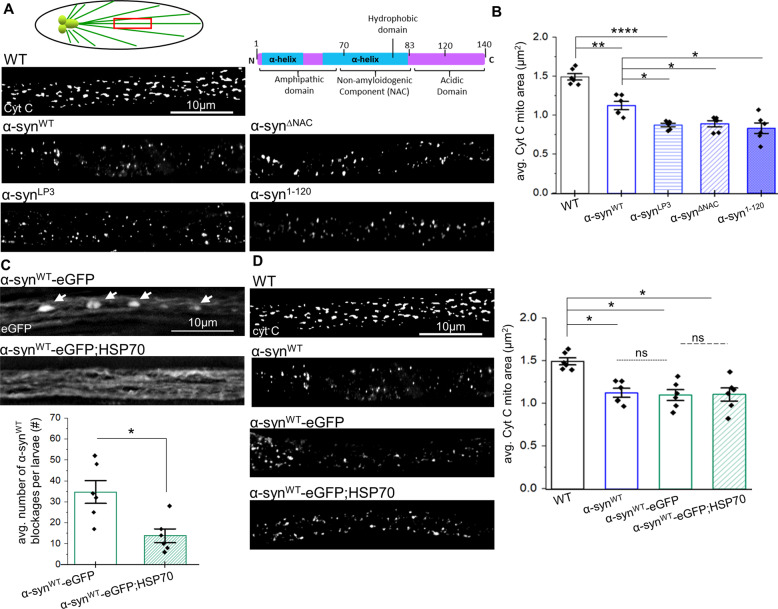
α-synuclein causes mitochondrial fragmentation independent of the C-terminus. **A** Schematic diagram of the larval nervous system. Schematic diagram of the 140aa α-syn showing the α-helical amphipathic domain, the non-amyloidogenic component region (NAC) and the acidic C-terminal region. Representative images from larval segmental nerves from wild type (WT) and larva expressing human α-syn^WT^, excess α-syn (α-syn^LP3^), α-syn^ΔNAC^ (deletion of aa71–82 (NAC), and α-syn^1–120^ (deletion of aa121–140) immunostained with the mitochondria marker cytochrome C (cyt C). Scale bar = 10 µm. **B** Quantification of the avg. mitochondria area (µm^2^) per larvae revealed the extent of mitochondrial fragmentation. Larvae expressing α-syn^WT^ (*p* *<* 0.001) and α-syn^LP3^ (*p* *<* 0.0001*)* showed a significant reduction in mitochondria areas compared to WT. Note that the avg. mitochondria area of α-syn^LP3^ larvae were found to be significantly decreased compared to α-synWT (*p* *<* 0.01). Further, larvae expressing α-syn^ΔNAC^ (*p* *<* 0.01) or α-syn^1–120^ (*p* *<* 0.01) also showed a significant reduction in mitochondria areas compared to α-sy^nWT^, similar to α-syn^LP3^. **C** Representative images from segmental nerves of larva expressing α-syn^WT^-eGFP alone or larva expressing α-syn^WT^-eGFP in the context of excess HSP70. Scale bar = 10 µm. Quantification of the avg. number of α-syn^WT^-eGFP blockages per larvae (#) revealed a significant reduction in blockages with excess HSP70 compared to α-syn^WT^-eGFP alone (*p* *<* 0.01). **D** Representative images from segmental nerves from WT and larva expressing α-syn^WT^, α-syn^WT^-eGFP, or α-syn^WT^-eGFP in the context of excess HSP70 that have been immunostained with cyt C. Scale bar = 10 µm. Quantification of the avg. mitochondria area (µm^2^) per larvae revealed that larvae expressing either α-syn^WT^-eGFP or α-syn^WT^-eGFP in the context of excess HSP70 were found to be significantly decreased compared to WT (*p* *<* 0.01) and similar to larva expressing α-syn^WT^ (ns). *n* = 6 larvae, >250 mitochondria. *S*tatistical significance was determined using the two-sample two-sided Student’s *t* test. *p < 0.01, **p < 0.001, ***p < 0.0001, ****p < 0.00001.

Common neuropathological features seen in both familial (fPD) and sporadic (sPD) forms of PD are neuronal loss and α-syn-rich Lewy bodies. However, since the presence of impaired mitochondrial function, fragmentation, and oxidative stress have also been reported [13], a key unanswered question is how α-syn affects mitochondrial homeostasis. To address this problem in a whole organism, we expressed wild-type (WT) human α-syn in *Drosophila* larval neurons using the pan-neuronal GAL4 driver, Appl-GAL4 and examined single mitochondria. Although the gene that codes for α-syn (SNCA) is absent in *Drosophila*, *Drosophila* models expressing either WT or PD-linked mutant forms of human α-syn replicate several features of PD including loss of dopaminergic neurons, Lewy body-like inclusions and locomotor dysfunction [14, 15], and have been used to isolate the mechanistic roles of α-syn function [14, 16–20]. We found that α-syn co-localized with mitochondria in *Drosophila* larval axons (Fig. S1A). Further, fragmented mitochondria were observed in segmental nerves from larvae expressing WT human α-syn (α-syn^WT^ or α-syn^WT^-eGFP) stained with the mitochondrial inner membrane (IMM) marker cytochrome C (cyt C) compared to control WT larvae (Fig. 1A). Enhanced α-syn expression (α-syn^LP3^, [21]) amplified the number of fragmented mitochondria observed (Fig. 1A, B). These results are consistent with previous observations [4, 6, 8–10] and suggest that the amount of α-syn expression correlates with the extent of mitochondrial fragmentation (Fig. 1A, B, S1B).

One mechanism that can cause mitochondrial fragmentation is α-syn aggregation. Previous work has suggested that α-syn aggregates/oligomers localizing to mitochondria causes fragmentation [22, 23]. To test this proposal, we examined larvae co-expressing HSP70 with α-syn^WT^, since Auluck et al. [20] showed that excess HSP70 prevented α-syn aggregation and DA neuron loss in fly brains. We also previously showed that excess HSP70 suppressed polyQ-mediated axonal blockages perhaps by modulating the soluble properties of the pathogenic polyQ protein, by preventing abnormal interactions with other proteins, or by rescuing chaperone depletion [24]. We found that while expression of α-syn^WT^ or α-syn^WT^-eGFP alone showed α-syn accumulations (Fig. 1C, [21]) and fragmented mitochondria (Fig. 1D), co-expression of HSP70 with α-syn^WT^-eGFP (Fig. 1D) failed to eliminate fragmented mitochondria, although the number of α-syn accumulations were rescued (Fig. 1C, D). Therefore, although α-syn and mitochondria co-localize, α-syn-mediated fragmentation is likely independent of α-syn aggregation. Further, since work has shown that deletion of aa71–82 in the NAC region (α-syn^ΔNAC^) eliminates α-syn aggregation [14, 21], while deletion of aa121–140 of the C-terminal domain (α-syn^1–120^) exacerbates α-syn accumulation [14], we next examined larvae expressing α-syn^ΔNAC^ or α-syn^1–120^. We found that while both α-syn^ΔNAC^ and α-syn^1–120^ express α-syn at the same level as α-syn^WT^ (Fig. S1B), mitochondrial fragmentation was not eliminated in these larval axons (Fig. 1A, B), indicating that neither the NAC region nor the C-terminus of α-syn are required for fragmentation (Fig. 1A, B). Therefore, while α-syn aggregation likely does not influence mitochondrial fragmentation, perhaps the biophysical properties exerted by the N-terminal region of α-syn on mitochondrial membranes contribute to the extent of fragmentation.

### α-syn-mediated mitochondrial fragments are damaged/oxidized and show biased retrograde motility

**Fig. 2:**
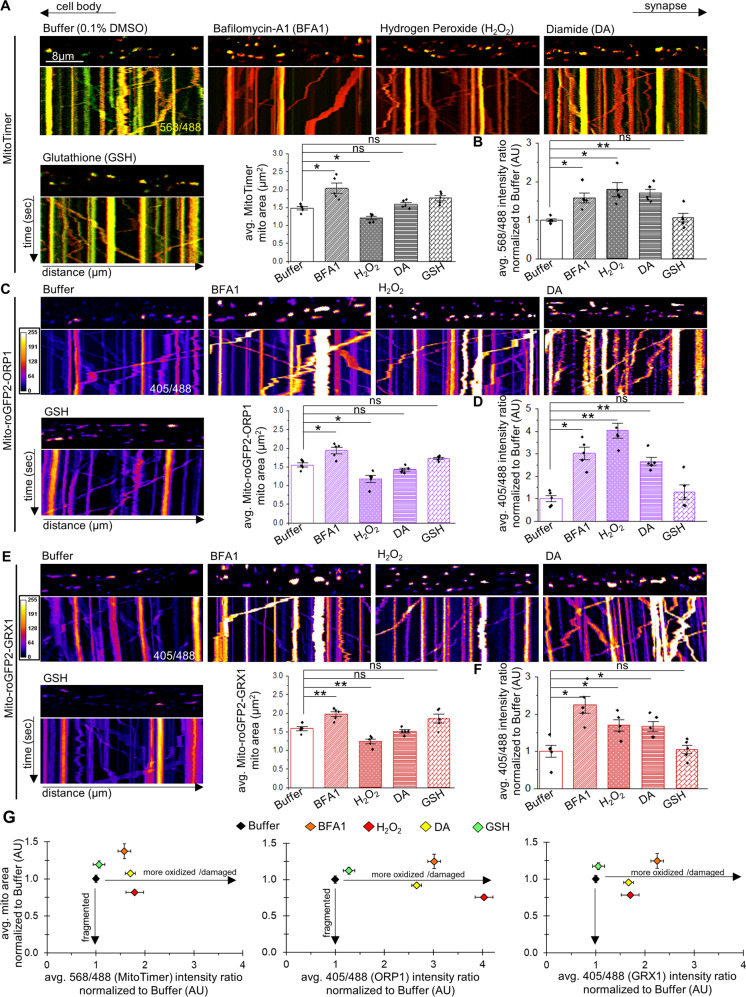
Oxidized or damaged mitochondria are not always fragmented in vivo. Representative images from merged movies and kymographs captured using simultaneous dual-view imaging of larval segmental nerves expressing either **A** MitoTimer (red: 568 nm and green: 488 nm), **C** Mito-roGFP2-ORP1 (405 and 488 nm), or **E** Mito-roGFP2-GRX1 (405 and 488 nm) that were fed fly food laced with buffer, 1 µM bafilomycin-A1 (BFA1), 25 mM hydrogen peroxide (H_2_O_2_), 5 mM diamide (DA), or 0.5 mM reduced glutathione (GSH) for 3 h prior to dissection and in vivo imaging. The horizontal arrows depict the direction of the cell body and synapse. X/Y axis on the kymograph depict time in seconds (s) and distance traveled in micrometers (µm). Scale bar = 8 µm. Quantification of the avg. mitochondria area (µm^2^) in larvae expressing **A** MitoTimer, **C** Mito-roGFP2-ORP1, or **E** Mito-roGFP2-GRX1 revealed significant increases with BFA1-treatment (*p* < 0.01, *p* < 0.01, *p* < 0.001, respectively), significant decreases with H_2_O_2_-treatment (*p* < 0.01, *p* < 0.01, *p* < 0.001, respectively), or no change with either DA- or GSH-treatment (ns) compared to larvae treated with buffer. Quantification of the intensity ratios (AU) from either **B** MitoTimer (red: 568 nm/green: 488 nm), **D** Mito-roGFP2-ORP1 (405/488 nm), or **F** Mito-roGFP2-GRX1 (405/488 nm) revealed significant increases in the relative intensity ratios (normalized to buffer-treated larvae) with either BFA1-treatment (*p* < 0.01, *p* < 0.01, *p* < 0.01, respectively), H_2_O_2_-treatment (*p* < 0.01, *p* < 00.01, *p* < 0.01, respectively), or DA-treatment (*p* < 0.001, *p* < 0.001, *p* < 0.01, respectively) compared to buffer-treated larvae, while GSH-treatment had no effect (ns). **G** Avg. mitochondria area normalized to WT (y-axis in AU) was compared to intensity ratios of either MitoTimer, Mito-roGFP2-ORP1, or Mito-roGFP2-GRX1 normalized to WT (AU) showed that oxidized/damaged mitochondria are not always fragmented. *n* = 5 larvae, >120 mitochondria. *S*tatistical significance was determined using the two-sample two-sided Student’s *t* test. ns = *p* > 0.01, *p < 0.01, **p < 0.001.

Several studies propose that mitochondrial fission/fragmentation is associated with mitochondrial dysfunction/oxidation [25, 26]. However, we found using the mitochondrial turnover reporter MitoTimer and the mitochondrial oxidation reporters Mito-roGFP2-ORP1 and Mito-roGFP2-GRX1 that old/damaged/oxidized mitochondria are not always fragmented in vivo (Figs. 2, S4, see Supplementary materials). First, the temporal and spatial characteristics of individual mitochondrial turnover in living neurons were examined under a variety of stress condition. Larvae expressing MitoTimer (Figs. S2, S3, S4), Mito-roGFP2-ORP1 or Mito-roGFP2-GRX1 (Fig. S5) in 8 motor neurons (pGAL4-62B SG26-1 [27, 28]) were exposed to cold-stress, heat-stress, mechanical-stress, elevated mitochondria fission (UAS-Drp1), elevated mitochondria fusion (UAS-hMFN2), inhibition of autophagy (Bafilomycin-A1—BFA1), excess chemical oxidants (hydrogen peroxide—H_2_O_2_, or Diamide—DA), or excess chemical reductants (glutathione reduced—GSH) (Figs. 2, S4). The changes in 568/488 nm ratios of MitoTimer (Figs. 2, S2, S4) or 488/405 nm ratios of Mito-roGFP2-ORP1/GRX1 (Figs. 2, S5, S6) indicate that these in vivo probes can monitor the health of individual mitochondria and that damaged/old/oxidized mitochondria are not always fragmented under physiological conditions (Table S1), supporting the proposition that mitochondrial oxidation is not always associated with mitochondrial fragmentation.

**Fig. 3:**
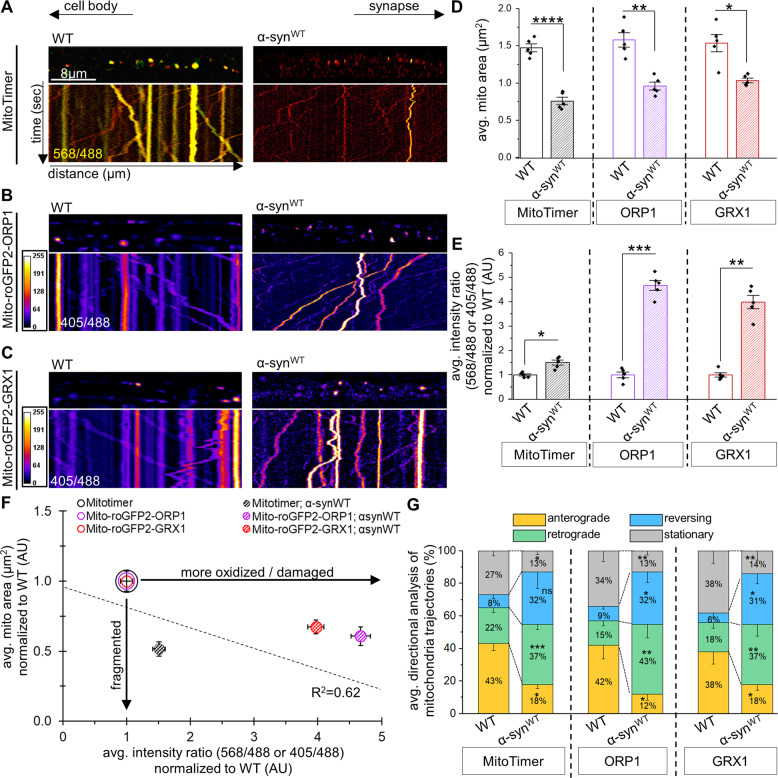
α-synuclein-mediated fragmented mitochondria are oxidized/damaged and show retrograde biased axonal movement. Representative larval segmental nerves and corresponding kymographs from merged movies captured using simultaneous dual-view imaging of larvae expressing either **A** MitoTimer, **B** Mito-roGFP2-ORP1, or **C** Mito-roGFP2-GRX1 alone or in the context of simultaneous α-synuclein expression (α-syn^WT^). The horizontal arrows depict the direction of the cell body and synapse. X/Y axis on the kymograph depict time in seconds (s) and distance traveled in micrometers (µm). Scale bar=8 µm. **D** Quantification of the average mitochondrial area (µm^2^) in larvae expressing α-syn^WT^ with either MitoTimer, Mito-roGFP2-ORP1, or Mito-roGFP2-GRX1 revealed significant decreases in mitochondria area (*p* < 0.00001, *p* < 0.001, *p* < 0.01, respectively) compared to WT. **E** Quantification of the intensity ratios (AU) from either MitoTimer (red: 568 nm/green: 488 nm), Mito-roGFP2-ORP1 (405/488 nm), or Mito-roGFP2-GRX1 (405/488 nm) showed significant increases with α-syn^WT^ expression (*p* < 0.01, *p* < 0.0001, *p* < 0.001, respectively) compared to WT. **F** Avg. mitochondria area normalized to WT (y-axis in AU) was compared to intensity ratios of either MitoTimer, Mito-roGFP2-ORP1, or Mito-roGFP2-GRX1 normalized to WT (AU) showed that syn^WT^-mediated fragmented mitochondria are more oxidized. Area and intensity ratios were paired to calculate a correlation coefficient across all genotypes, with a line of best fit drawn to represent the correlation between the average mitochondria area and the average intensity ratios. The correlation coefficient *R*^2^ = 0.62. **G** Quantification of mitochondrial movement directionality (%) in larvae expressing α-syn^WT^ with either MitoTimer, Mito-roGFP2-ORP1, or Mito-roGFP2-GRX1 revealed significant decreases in anterogradely moving mitochondria (*p* < 0.01, *p* < 0.01, *p* < 0.01, respectively) and significant increases in retrogradely moving mitochondria (*p* < 0.0001, *p* < 0.001, *p* < 0.001, respectively) compared to WT. Larvae expressing α-syn^WT^ also showed a significant decrease in the percentage of stationary mitochondria (*p* < 0.01, *p* < 0.01, *p* < 0.01, respectively). *n* = 5 larvae, >120 mitochondria. Statistical significance was determined using the two-sample two-sided Student’s *t* test. *p < 0.01, **p < 0.001, ***p < 0.0001, ****p < 0.00001.

To test whether α-syn-mediated fragmented mitochondria are damaged, we examined the health/oxidation of individual mitochondria in larvae expressing α-syn^WT^ with MitoTimer (Fig. 3A), Mito-roGFP2-ORP1 (Fig. 3B) or Mito-roGFP2-GRX1 (Fig. 3C). α-syn^WT^-mediated fragmented mitochondria were predominantly red with MitoTimer (Fig. 3A–E) or yellow/white with Mito-roGFP2-ORP1/GRX1 (Fig. 3B, C, E), with a high correlation coefficient (*R*^2^ = 0.62, Fig. 3F) between mitochondrial fragmentation (Fig. 3D) and oxidation/damage (Fig. 3E), indicating that under physiological conditions α-syn-mediated mitochondrial fragments are likely damaged/old and highly oxidized.

Since coordinated cycles of fission-fusion together with motility maintain mitochondrial shape, we next evaluated mitochondrial motility behaviors in the context of α-syn. Under normal conditions, MitoTimer/Mito-roGFP2 labeled mitochondria move bi-directionally with an anterograde bias (Fig. 3G). However, expression of α-syn^WT^ shifted the overall mitochondrial motility distribution to a more retrograde bias with significant decreases seen in anterogradely moving and stalled mitochondrial populations (Fig. 3G). Therefore, α-syn-mediated fragmented and damaged mitochondria are likely transported retrogradely and are perhaps destined for mitophagy at the cell bodies [6, 29].

### The C-terminal of α-syn is essential for mitochondrial oxidation and for retrograde biased motility

**Fig. 4:**
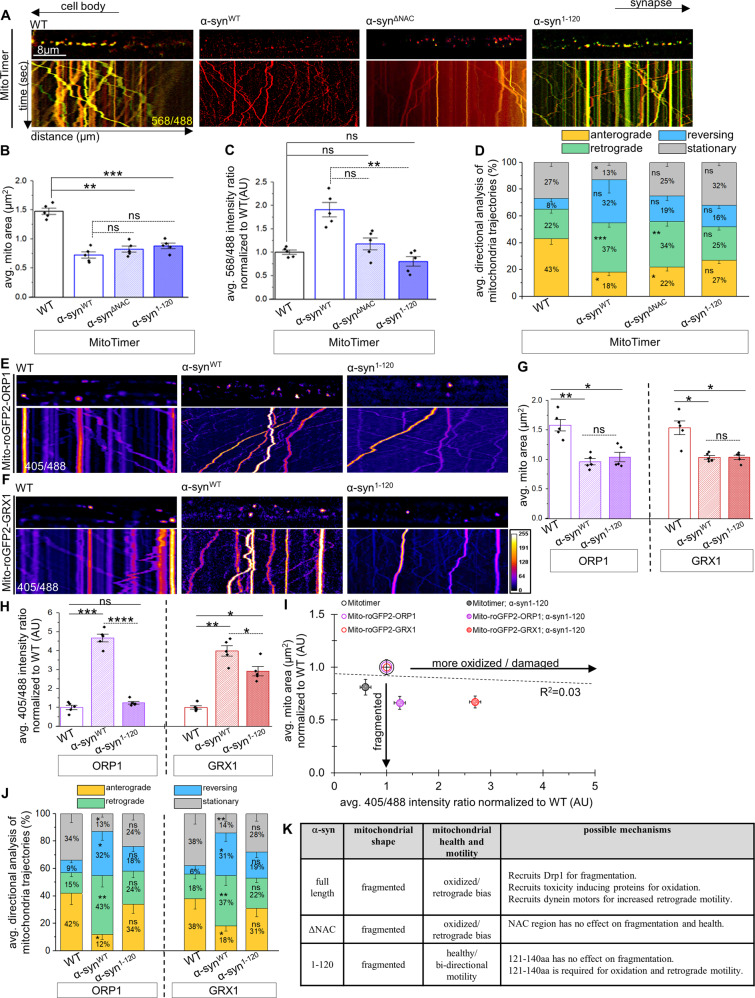
The C-terminus of α-syn is required to stimulate oxidation and the retrograde motility bias of mitochondria. **A** Representative larval segmental nerves and corresponding kymographs from merged movies captured using simultaneous dual-view imaging of larvae expressing MitoTimer alone or simultaneous with either αsyn^WT^, αsyn^ΔNAC^, or αsyn^1–120^. The horizontal arrows depict the direction of the cell body and synapse. X/Y axis on the kymograph depict time in seconds (s) and distance traveled in micrometers (µm). Scale bar = 8 µm. **B** Quantification of the avg. mitochondria area (µm^2^) reported by MitoTimer revealed significant decreases in larvae expressing α-syn^ΔNAC^ (*p* < 0.001) or α-syn^1–120^ (*p* < 0.0001) compared to WT, which are similar (ns) to larvae expressing α-syn^WT^. **C** Quantification of the intensity ratios (AU) from MitoTimer (red: 568 nm/green: 488 nm) revealed significant decrease in the avg. intensity ratio of larvae expressing α-syn^1–120^ compared to larvae expressing α-syn^WT^ (*p* < 0.001), which were similar to WT (ns). Larvae expressing α-syn^ΔNAC^ and larvae expressing α-syn^WT^ are not significantly different (ns). **D** Quantification of mitochondria movement directionality (%) in larvae expressing α-syn^1–120^ showed similar populations of anterograde (ns), retrograde (ns), stationary (ns), and reversing (ns) mitochondria as WT, while larvae expressing α-syn^ΔNAC^ showed a cargo population distribution similar to larvae expressing α-syn^WT^, with decreased anterograde populations (*p* < 0.01) and increase retrograde populations (*p* < 0.001) of mitochondria compared to WT. **E**, **F** Representative larval segmental nerves and corresponding kymographs from merged movies captured using simultaneous dual-view imaging of larvae expressing either Mito-roGFP2-ORP1 or Mito-roGFP2-GRX1 alone or simultaneously with either αsyn^WT^ or αsyn^1–120^. Scale bar = 8 µm. **G** Quantification of the avg. mitochondria area (µm^2^) reported by Mito-roGFP2-ORP1 or Mito-roGFP2-GRX1 revealed significant decreases in area of larvae expressing α-syn^1–120^ (*p* < 0.01, *p* < 0.01, respectively) compared to WT, which are similar to mitochondria area from larvae expressing α-syn^WT^ (ns). **H** Quantification analysis of 405/488 intensity ratios (AU) of Mito-roGFP2-ORP1 or Mito-roGFP2-GRX1, normalized to WT, revealed significant decreases in the avg. intensity ratio of larvae expressing α-syn^1–120^ compared to larvae expressing α-syn^WT^ (*p* < 0.00001, *p* < 0.01 respectively). Note intensity ratios of Mito-roGFP2-ORP1 larvae co-expressing α-syn^1–120^ were not different (ns) from WT, while intensity ratios from Mito-roGFP2-GRX1 larvae co-expressing α-syn^1–120^ were significantly increased (*p* < 0.01) compared to WT. **I** Avg. mitochondria area normalized to WT (AU) was compared to intensity ratios normalized to WT (AU) of larvae expressing MitoTimer, Mito-roGFP2-ORP1, or Mito-roGFP2-GRX1 either alone or with α-syn^1–120^ showed that mitochondria are fragmented but are not highly oxidized/damaged. Area and intensity ratios were paired to calculate a correlation coefficient across all genotypes, with a line of best fit drawn. Correlation coefficient *R*^2^ = 0.03. **J** Quantification of mitochondria movement directionality (%) in larvae expressing α-syn^1–120^ showed similar populations of anterograde (ns), retrograde (ns), stationary (ns), and reversing (ns) mitochondria as WT in either Mito-roGFP2-ORP1 or Mito-roGFP2-GRX1 larvae, similar to observations with larvae co-expressing MitoTimer and α-syn^1–120^ (**D**). **K** Table illustrating an overview of the findings from panels (**A**) to (**J**). *n* = 5 larvae, >120 mitochondria. Statistical significance was determined using the two-sample two-sided Student’s *t* test. ns = *p* > 0.01, *p < 0.01, **p < 0.001, ***p < 0.0001, ****p < 0.00001.

We next examined if different regions of α-syn affect mitochondrial health. Consistent with Fig. 1, larvae co-expressing α-syn^ΔNAC^ or α-syn^1–120^ with MitoTimer, Mito-roGFP2-ORP1, or Mito-roGFP2-GRX1 showed fragmented mitochondria compared to WT larvae (Fig. 4A, B, E, F, G). While larvae expressing α-syn^ΔNAC^ were comparable to α-syn^WT^, both showing damaged/oxidized mitochondria, in contrast α-syn^1–120^ larvae showed more healthy mitochondria similar to WT, with significantly decreased 568/488 nm or 405/488 nm intensity ratios compared to larvae expressing α-syn^WT^ (Fig. 4A, C, E, F, H), indicating that truncation of the C-terminus of α-syn rescues mitochondrial damage. Correlative analysis of the average mitochondrial area (normalized to WT) vs the average intensity ratio (568/488 nm or 405/488 nm normalized to WT) (Fig. 4I) showed that unlike with full length α-syn (Fig. 3F), deletion of the C-terminus weakens the α-syn-mediated link between mitochondrial fragmentation and mitochondrial damage/oxidation (*R*^2^ = 0.03). Therefore, the C-terminus of α-syn likely mediates mitochondrial oxidation independent of fragmentation (Fig. 4K, Table S2).

Further, we found that the retrograde bias seen in α-syn^WT^ was also rescued, with a significant decrease in the percentage of retrogradely moving mitochondria in larvae expressing α-syn^1–120^ compared to larvae expressing α-syn^WT^. The decrease in retrogradely moving mitochondria was due to significant increases in the anterogradely moving and stalled mitochondrial populations (Fig. 4D–J). In contrast, similar to α-syn^WT^, larvae expressing α-syn^ΔNAC^ still showed a retrograde bias (Fig. 4D–J). Therefore, the C-terminus of α-syn likely mediates the retrograde motility of oxidized mitochondria (Fig. 4K, Table S2).

### α-syn-mediated mitochondrial fragmentation is DRP1-dependent

**Fig. 5:**
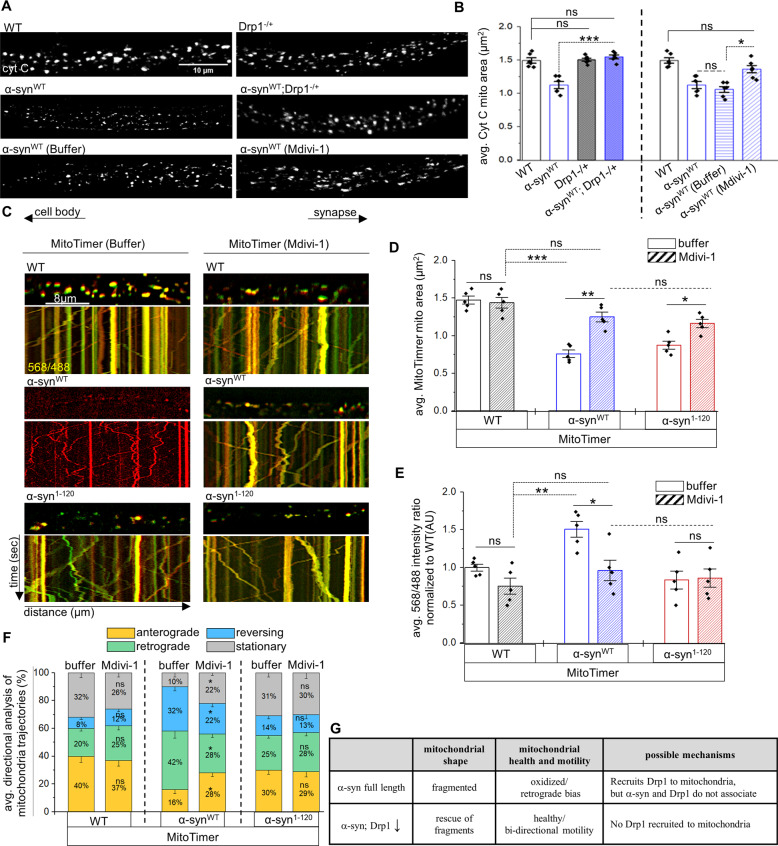
Mdivi-1-mediated Drp1 inhibition rescues α-synuclein-mediated fragmentation and oxidation independent of the C-terminus of α-syn. **A** Representative nerve images of WT larvae, larvae expressing α-syn^WT^, larvae containing a heterozygous reduction of Drp1 (Drp1^KG03815/+^), larvae expressing α-syn^WT^ in the context of DRP^+/−^, larvae expressing α-syn^WT^ fed food containing buffer for 8 h, or larvae expressing α-syn^WT^ fed food laced with 10 µM Mdivi-1 for 8 h and immunostained with cyt C. Scale bar = 10 µm. **B** Quantification of mitochondria area (µm^2^) revealed that expression of α-syn^WT^ in the context of Drp1^+/−^ significantly increased mitochondria areas compared to larvae expressing α-syn^WT^ alone (*p* < 0.0001), and are now similar to WT (ns). Note, Drp1^+/−^ larvae were similar to WT (ns). Further quantification revealed that feeding α-syn^WT^ expressing larvae food laced with 10 µM Mdivi-1 for 8 h significantly increased mitochondria areas compared to buffer fed larvae expressing α-syn^WT^ (*p* < 0.01). Mdivi-1 fed larvae expressing α-synWT showed similar mitochondria areas as WT (ns). Note that buffer fed α-synWT expressing larvae have mitochondria similar to α-synWT larvae fed on normal food (ns). *n* = 6 larvae, >250 mitochondria. **C** Representative larval segmental nerves and corresponding kymographs from merged movies captured using simultaneous dual-view imaging of larvae expressing MitoTimer alone or co-expressing MitoTimer with either α-syn^WT^ or α-syn^1–120^ that have been fed fly food laced with buffer for 8 h or fly food laced with 10 µM Mdivi-1 for 8 h prior to dissection and in vivo imaging. The horizontal arrows depict the direction of the cell body and synapse. X/Y axis on the kymograph depict time in seconds (s) and distance traveled in micrometers (µm). Scale bar = 8 µm. **D** Quantification of average mitochondria area reported by MitoTimer revealed that larvae expressing α-syn^WT^ fed on 10 µm Mdivi-1 showed mitochondria areas that are comparable to larvae expressing MitoTimer fed on buffer (ns). However, mitochondria areas were significantly increased in these larvae compared to larvae expressing α-syn^WT^ fed on buffer (*p* < 0.001). Further analysis revealed that larvae expressing α-syn^1–120^ fed food laced with 10 µM Mdivi-1 showed a significant increase in the average mitochondria area compared to larvae expressing α-syn^1–120^ fed on buffer laced food (*p* < 0.01), which are not different from mitochondria areas of α-synWT fed food laced with 10 µM Mdivi-1 (ns). **E** Quantification of the intensity ratios (AU) from MitoTimer (red: 568 nm/green: 488 nm) revealed that feeding 10 µM Mdivi-1 to larvae expressing α-syn^WT^ significantly decreased the red/green intensity ratio (*p* < 0.01) compared to larvae expressing α-syn^WT^ that were fed on buffer. Further analysis revealed that larvae expressing α-syn^1–120^ fed on 10 µM Mdivi-1 are similar to larvae expressing α-syn^1–120^ fed on buffer (ns), which is also similar to α-syn^WT^ larvae fed food laced with 10 µM Mdivi-1 (ns). **F** Quantification of the mitochondria movement directionality (%) in larvae expressing α-syn^WT^ fed on 10 µM Mdivi-1 showed a significant decrease in retrograde moving mitochondria (*p* < 0.01), a significant increase in the anterogradely moving mitochondria (*p* < 0.01), a significant decrease in the reversing population of mitochondria (*p* < 0.01), and a significant increase in the stationary population of mitochondria (*p* < 0.01) compared to buffer fed α-syn^WT^ expressing larvae. No significant changes in the mitochondrial populations between larvae expressing α-syn^1–120^ fed on 10 µM Mdivi-1 or buffer laced food were observed (ns), similar to WT larvae (ns). **G** Table illustrating an overview of the findings from panels (**A**) to (**F**). *n* = 5 larvae, >120 mitochondria. Statistical significance was determined using the two-sample two-sided Student’s *t* test. ns = *p* > 0.01, *p < 0.01, **p < 0.001, ***p < 0.0001.

One putative mechanism for how α-syn can induce mitochondrial fragmentation is by influencing the function of mitochondrial fission-fusion proteins. Mutations in DRP1 block fission, causing elongated mitochondria, while excess DRP1 increases mitochondrial fission causing fragmented mitochondria ([4, 8, 11], Fig. S8A). Homozygous DRP1 mutants (Drp1^KG03815^) are lethal with larvae pupating but do not enclose to adults. These homozygous DRP1 mutant larvae show neurophysiology defects with elongated mitochondria [30], while heterozygous DRP1 mutant larvae show normal mitochondrial morphology (Fig. 5A, S8A). In contrast, MFN mutants block fusion, causing small rounded mitochondria [31], while excess MFN cause mitochondrial elongation ([32], Fig. S8A). We found that expression of α-syn^WT^ in the context of DRP1 reduction resulted in normal mitochondria that were comparable to WT (Fig. 5A, B). Further, while WT larvae fed high concentrations of the mitochondrial division inhibitor-1 (Mdivi-1), a cell-permeable selective inhibitor of DRP1 (≥500 µM) were lethal, heterozygous DRP1 mutant larvae that were fed Mdivi-1 at 10 µM (8 h) showed elongated mitochondria (Fig. 5A). Similar to genetic reduction of DRP1, larvae expressing α-syn^WT^ that were fed Mdivi-1 at 10 µM also showed mitochondrial sizes that were comparable to WT (Fig. 5A, B), indicating rescue of mitochondrial fragmentation. Therefore, α-syn-mediated mitochondrial fragmentation is likely dependent on DRP1.

Further, larvae co-expressing α-synWT and DRP1 also showed mitochondrial sizes comparable to WT mitochondria (Fig. S8A). In contrast however, larvae co-expressing α-synWT and MFN2 had no effect on α-syn-mediated fragmentation (Fig. S8A). Since changing the dose of DRP1-rescued α-syn-mediated mitochondrial fragmentation, it is possible that α-syn and DRP1 play compensatory roles during mitochondrial shaping, perhaps via a mechanism where excess α-syn and/or excess DRP1 sequesters each other away from mitochondria. However, while we cannot eliminate transient interactions, we did not observe α-syn and DRP1 directly associating with each other in co-IP experiments (Fig. S8B). Perhaps α-syn instigates DRP1-mediated events on mitochondrial membranes by altering the intracellular localization of DRP1 ([33], Fig. 5G).

To determine the health of DRP1-rescued mitochondria in α-syn expressing larvae, we next assayed mitochondrial turnover with MitoTimer in the context of Mdivi-1 and α-syn since we were unable to genetically reduce DRP1 while simultaneously co-expressing α-syn^WT^ and MitoTimer in the same larvae. In control experiments, elongated mitochondria were seen in Mdivi-1-fed Drp1^KG03815^ heterozygous larvae expressing MitoTimer, while buffer fed larvae were comparable to WT (Fig. S9). In contrast, Mdivi-1-fed larvae co-expressing α-syn^WT^ and MitoTimer showed normal sized mitochondria that were healthier/less damaged with a significant decrease in the 568/488 nm intensity ratio compared to buffer-treated α-syn^WT^; MitoTimer larvae which were fragmented and damaged (Fig. 5C, D, E). These results are consistent with a previous report that showed that Mdivi-1 was neuroprotective in an A53T α-syn PD rat model [34]. Further, the α-syn-mediated retrograde mitochondrial motility bias was also rescued in Mdivi-1-fed α-syn^WT^ larvae compared to buffer-treated α-syn^WT^ larvae (Fig. 5F). Therefore, while modulating DRP1 levels rescues α-syn-mediated mitochondrial damage, we propose that the DRP1-mediated α-syn-induced mitochondrial fragmentation is likely upstream of α-syn-induced mitochondrial oxidation/damage and motility changes.

Further, since deletion of the C-terminus rescued α-syn-mediated mitochondrial oxidation and retrograde bias, but not fragmentation (Figs. 1, 4, Table S2), we propose that fragmentation is likely mediated by the N-terminus of α-syn. Therefore, inhibiting DRP1 in the context of α-syn^1–120^ should also rescue α-syn-mediated fragmentation. Indeed, feeding Mdivi-1 to larvae co-expressing α-syn^1–120^;MitoTimer rescued mitochondrial fragmentation in contrast to buffer fed larvae (Fig. 5C, D). However, similar to mitochondria seen in buffer fed α-syn^1–120^;MitoTimer larvae, the 568/488 nm intensity ratio was unaltered (Fig. 5C, E) and no significant changes to mitochondrial motility were seen in Mdivi-1 fed α-syn^1–120^;MitoTimer larvae (Fig. 5F). Therefore, we propose that the N-terminal region of α-syn likely mediates events on mitochondrial membranes via a DRP1-dependent pathway, and that mitochondrial fragmentation may precede α-syn-induced mitochondrial oxidation and the retrograde motility bias we observe (Table S2).

### Excess PINK1/Parkin rescues α-syn-mediated mitochondrial fragmentation, membrane depolarization, and neuronal cell death

**Fig. 6:**
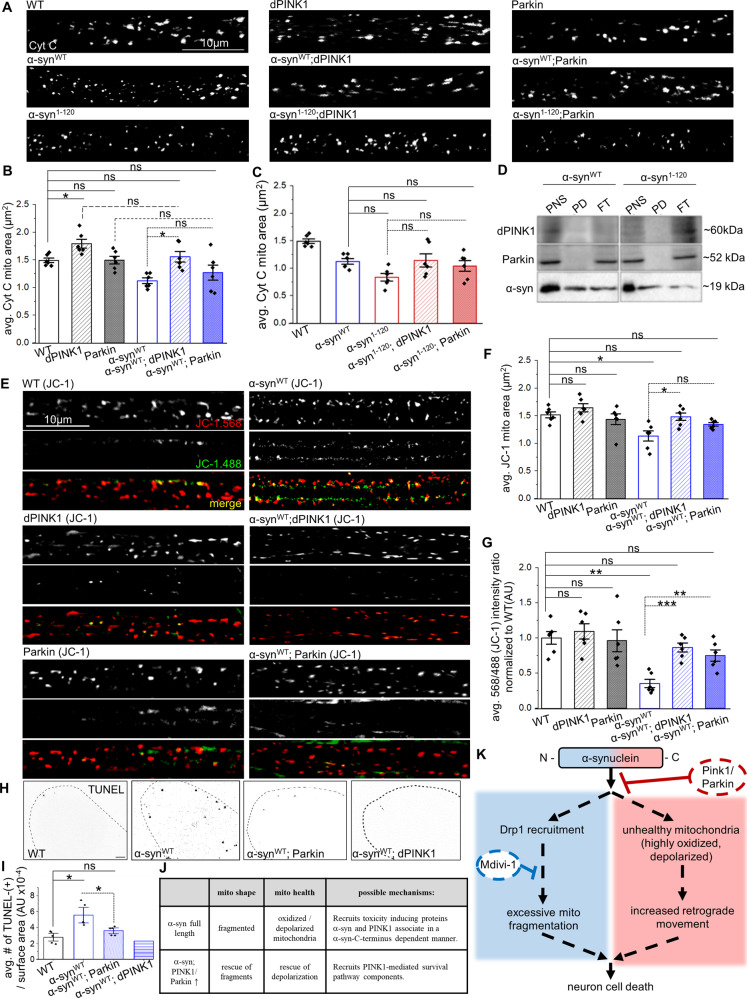
Excess dPINK1 or Parkin rescues α-synuclein-mediated mitochondrial fragmentation and damage, and neuronal cell death in a C-terminus-dependent manner. **A** Representative nerve images of WT larvae or larvae expressing either α-syn^WT^, α-syn^1–120^, UAS-dPINK1, UAS-Parkin, α-syn^WT^ with UAS-dPINK1, α-syn^WT^ with UAS-Parkin, α-syn^1–120^ with UAS-dPINK1, or α-syn^1–120^ with UAS-Parkin that are immunostained with cyt C. Scale bar = 10 µm. **B** Quantification of mitochondria area (µm^2^) revealed that simultaneous expression of α-syn^WT^ with UAS-dPINK1 showed mitochondria areas comparable to WT larvae (ns), which are significantly increased (*p* < 0.01) compared to α-syn_WT_ alone. Note, larvae co-expressing UAS-Parkin with α-syn^WT^ are not different (ns) from either WT or α-syn^WT^ larvae. **C** Quantification of mitochondria area (µm^2^) revealed that simultaneous expression of α-syn^1–120^ with UAS-dPINK1 or UAS-Parkin showed mitochondria areas that are also comparable to α-syn^WT^ larvae (ns). **D** Immunoprecipitation of α-synuclein (BD Biosciences, 1:1000) was performed on total PNS extracted from adult fly brains expressing either α-syn^WT^ or α-syn^1–120^, which shows α-synuclein immunoprecipitated in each case. α-synuclein immunoprecipitations were also co-stained for dPINK1 (Yang et al. [70]) or Parkin (Greene et al. [77]), *n* = 3. **E** Representative nerve images of WT larvae or larvae expressing either α-syn^WT^, UAS-dPINK1, UAS-Parkin, α-syn^WT^ with UAS-dPINK1, or α-syn^WT^ with UAS-Parkin, that are stained with JC-1 dye (Cayman Chemical, 1:800) for 10 min prior to dissection and in vivo imaging. Note, green staining represents JC-1 aggregates indicative of damaged mitochondria while red staining represented JC-1 monomers. Scale bar = 10 µm. **F** Quantification of mitochondria area (µm^2^) revealed that larva co-expressing α-syn^WT^ with UAS-dPINK1 showed mitochondria areas comparable to WT larvae (ns), which are significantly increased (*p* < 0.01) compared to α-syn^WT^ alone. Note, larvae co-expressing UAS-Parkin with α-syn^WT^ are not different (*ns*) from either WT or α-syn^WT^ larvae. **G** Quantification of the average red (568 nm)/green (488 nm) intensity ratio normalized to WT (AU) reported by JC-1 staining revealed that larvae expressing α-syn^WT^ exhibit a significantly decreased red/green intensity ratio (*p* < 0.001) compared to WT larvae. Further analysis revealed that larvae co-expressing α-syn^WT^ with either UAS-dPINK1 or UAS-Parkin showed red/green intensity ratios that are comparable to WT (ns) and which are significantly increased compared to α-syn^WT^ alone (*p* < 0.0001, *p* < 0.001, respectively). *n* = 5 larvae, >120 mitochondria. Statistical significance was determined using the two-sample two-sided Student’s *t* test. ns = *p* > 0.01, *p < 0.01, **p < 0.001, ***p < 0.0001. **H** Representative images from larval brains immunostained for TUNEL from WT larvae or larva expressing either α-syn^WT^ alone or α-syn^WT^ in the context of excess Parkin or dPINK1 show TUNEL-positive cells with α-syn^WT^ expression which are diminished with simultaneous expression of either excess Parkin or dPINK1. **I** Quantification of the number of TUNEL-positive cells per surface area (AU) revealed that α-syn^WT^ expression causes a significant increase (*p* < 0.05), while α-syn^WT^ expression in the context of excess Parkin was similar to WT (ns), and significantly decreased compared to larvae expressing α-syn^WT^ alone (*p* < 0.05). *n* = 5 larvae. Statistical significance was determined using the two-sample two-sided Student’s *t* test. ns = *p* > 0.05, *p < 0.05. **J** Table illustrating an overview of the findings from panels (**A**)–(**I**). **K** Flow chart illustrating an overview model by which the N- and C-terminus of α-syn make distinct associations to facilitate mitochondrial dynamics and quality control.

One mechanism by which α-syn could cause damaged/old and oxidized mitochondria is by directly affecting the mitochondrial quality control machinery. PINK1/Parkin work in conjunction to promote mitochondrial quality control [35] and turnover by mediating mitophagy [36]. PINK1 accumulates at damaged mitochondria where it functions as a kinase for Parkin translocation to depolarized mitochondria [37], and facilitates the mitophagy-mediated degradation of damaged mitochondria [38]. Since oxidative stress changes the mitochondrial membrane permeability and decreases the mitochondrial membrane potential (ΔΨm) [39], and depolarized mitochondria are thought to undergo mitophagy via a PINK1/Parkin-mediated mechanism [37], we tested the proposal that excess PINK1/Parkin will rescue α-syn-mediated mitochondrial oxidation. To this end, we first measured mitochondrial depolarization using JC-1, as we were unable to simultaneously express α-syn^WT^ and PINK1/Parkin with MitoTimer/Mito-roGFP2 in the same larvae. In WT controls, a steady-state population of red and yellow JC-1 labeled mitochondria were observed, while α-syn^WT^ larvae showed a robust increase in green mitochondria (Fig. 6E, G), indicating that α-syn-mediated mitochondrial fragments were depolarized. Note that α-syn^ΔNAC^ larvae were similar to α-syn^WT^ (Fig. S10A), while α-syn^1–120^ larvae showed significantly less depolarized mitochondria compared to α-syn^WT^ (Fig. S10A). Further, biochemical analysis showed that with excess α-syn^WT^ a significant amount of Cyt C was released from the heavy membrane to the cytosol, which is a key initial step in the apoptotic processes (Fig. S10B). In contrast, the ratio of sol/HM Cyt C released in α-syn^1–120^ was similar to WT (Fig. S10B). Therefore, these observations validate our proposal that the depolarization of mitochondrial membrane potential is likely dependent on the C-terminus of α-syn.

We next tested how excess PINK1/Parkin affects α-syn-mediated mitochondrial size. Co-expression of PINK1/Parkin with α-syn^WT^ decreased α-syn-mediated mitochondrial fragmentation, with PINK1 showing a significant rescue compared to α-syn^WT^ (Fig. 6A, B), supporting that PINK1 acts upstream and recruits Parkin to mitochondria [38]. Since the C-terminus of α-syn has been implicated in multiple protein interactions [40, 41], the PINK1-mediated rescue of α-syn-mediated mitochondrial fragmentation could result through direct associations between PINK1 and the α-syn C-terminus. Consistent with this proposal, expression of PINK1/Parkin failed to rescue α-syn-mediated mitochondrial fragmentation in α-syn^1–120^ (Fig. 6A, C), indicating that truncation of the α-syn C-terminus likely disrupts the translocation of PINK1/Parkin to damaged mitochondria. Indeed, deletion of α-syn aa121–140 abolished the α-syn-PINK1 association as assayed by Co-IP analysis (Fig. 6D). Therefore, while there is likely a functional interplay between PINK1/Parkin during mitochondrial fission and turnover, the C-terminus of α-syn plays a key role in mitochondrial quality control.

Further, excess PINK1/Parkin also prevented α-syn-mediated mitochondrial depolarization as demonstrated by a significant increase in the 568/488 nm JC-1 intensity ratio in α-syn^WT^; PINK1/Parkin larvae, which was similar to WT controls (Fig. 6E, G). Excess PINK1/Parkin also protected neurons from *α*-syn-induced cell death (Fig. 6H, I). Therefore, we propose that the α-syn C-terminus associates with PINK1/Parkin to rescue α-syn-mediated depolarization of mitochondrial fragments and prevent *α*-syn-induced neuronal cell death (Fig. 6J, Table S3).

## Discussion

Despite studies showing α-syn localization to mitochondria in many different model systems, the mechanistic details of how excess α-syn affects mitochondrial homeostasis during PD pathology in neurons in vivo remains elusive. Our study unravels currently unknown roles for α-syn in mitochondrial homeostasis at the level of a single mitochondrion in vivo, in a whole organism. Our observations suggest that [1] α-syn-mediated mitochondrial fragmentation is independent of α-syn aggregation properties, [2] the N-terminus of α-syn likely plays a role in mitochondrial fragmentation via a DRP1-dependent mechanism, and [3] the C-terminus of α-syn is required for A: mitochondrial oxidation/damage via PINK1/Parkin-mediated mitochondrial turnover, and B: the retrograde motility of oxidized mitochondria for mitophagy (Table S1–3). We propose a model in which specific regions of α-syn affect mitochondrial dynamics and quality control processes (Fig. 6K). Further, we identify that mitochondrial fragmentation is likely upstream of oxidation during α-syn-induced mitochondrial dysfunction on single mitochondria. Therefore, collectively, we have uncovered a common pathological pathway for PD, highlighting how mitochondrial dynamics and quality control pathways can be targeted for therapeutics, early before neuronal loss or clinical manifestation of α-syn-mediated neurodegeneration.

### The physiological relevance of the N-terminus of α-syn during mitochondrial fission

Consistent with our findings, many other studies done in many different model systems ranging from zebrafish to mice, both under in vivo and in vitro conditions have shown that expression of α-syn in neurons cause mitochondrial fragmentation [4, 6, 8–10]. Furthermore, selective targeting of α-syn to mitochondria in human neurons also induced mitochondrial fragmentation [6, 29]. However, a recent study by Ordonez et al. [42] showed that α-syn expression caused elongated mitochondria. This discrepancy may be due to the high levels of α-syn being expressed in this new *Drosophila* α-synucleinopathy model which showed severe phenotypes, such as cortical and neuropil vacuole formation, loss of neurons and caspase activation much earlier than previously reported in the α-syn model we used [14, 16–20]. Further, although α-syn lacks a true mitochondrial localization signal, α-syn is present in the outer mitochondrial membrane (OMM), inner mitochondrial membrane (IMM), mitochondrial matrix, and mitochondrial associated membranes (MAM [29], indicating that α-syn can directly affect mitochondrial functions. Since both deletion of NAC (α-syn^ΔNAC^) or truncation of the C-terminus (α-syn^1–120^) failed to eliminate mitochondrial fragmentation, we postulate that the N-terminus of α-syn likely plays a key functional role during mitochondrial morphology (Fig. 1, Table S2). Indeed, the N-terminus of α-syn is positively charged and is important for α-syn-lipid interactions [43]. Work has also shown that α-syn can bind many different lipids by adopting an N-terminal helical structure [1, 43, 44]. Further, the first 32aa of α-syn has been proposed to be essential for mitochondrial localization [11], with deletion of aa1–11 completely suppressing the in vitro binding of exogenous α-syn to isolated mitochondria [45]. Therefore, the structural conformation of the N-terminus of α-syn may mediate functional interactions with mitochondria, with excess α-syn causing adverse effects to mitochondrial morphology. Further analysis will be needed to confirm predictions of this proposal.

Alternatively, since α-syn can adopt β-sheet conformations which are associated with α-syn aggregation, Lewy body formation, and neurotoxicity [46–48], perhaps α-syn aggregates contribute to mitochondrial defects. However, we found that fragmentation is likely independent of α-syn aggregation, since preventing aggregation in α-syn^ΔNAC^ (Fig. 1), which lacks HMW α-syn [21], or co-expressing HSP70 with α-syn, which suppressed aggregate formation (Fig. 1C, [20]) failed to eliminate mitochondrial fragmentation. Therefore, the α-syn-mediated mitochondrial morphology defects we observed are unlikely to be a consequence of aggregated α-syn in vivo.

α-syn could modulate the role of mitochondrial shaping proteins. Indeed, reduction of DRP1-rescued α-syn-mediated mitochondrial fragmentation (Fig. 5, Table S2). However, excess DRP1 but not excess MFN2 also rescued α-syn-mediated fragments (Fig. S8A, Table S1). At least two possible mechanisms could explain how α-syn can affect mitochondrial morphology, which are perhaps not mutually exclusive; 1: α-syn has a role in mitochondrial fragmentation independent of DRP1, and/or 2: α-syn and DRP1 play interdependent and coordinated roles during mitochondrial fission. Indeed, several studies support a role for α-syn in mitochondrial fragmentation independent of DRP1. Work showed that α-syn caused fragmentation in the absence of DRP1 [4]. Force delivery of α-syn to mitochondrial membranes led to mitochondrial fragmentation [6]. Intriguingly, endogenous α-syn levels also resulted in mitochondrial fragmentation [8]. Taken together these observations hint at a possible role for α-syn in mitochondrial shaping independent of DRP1.

Alternatively, α-syn and DRP1 may have interdependent and coordinated roles during mitochondrial fission. Indeed, work has shown that excess α-syn can increase DRP1 translocation to mitochondria [49]. Perhaps changing the balance in the dose of either α-syn and DRP1 cause changes to mitochondrial shaping events. Indeed, DRP1 depletion by either the DRP1 null mutant or with Mdivi treatment (Fig. 5, Table S2) rescued mitochondrial fragmentation caused by excess α-syn. However, the fact that excess DRP1 also rescued excess α-syn-mediated mitochondrial fragmentation (Fig. S8, Table S1) may suggest that α-syn and DRP1 play compensatory roles in mitochondrial shaping, perhaps via a mechanism where excess α-syn and/or excess DRP1 sequesters each other away from mitochondria, mitigating changes in the balance of shaping proteins and preventing mitochondrial fission. Although we did not observe α-syn and DRP1 directly associating with each other in our co-IP experiments (Fig. S8B), we cannot rule out the possibility that α-syn-DRP1 interactions are transient. Indeed, several reports indicate that DRP1-receptor interactions are of low-affinity or transient, and can only be detected using cross-linking reagents [50, 51]. Alternatively, unidentified proteins could also mediate α-syn-DRP1 associations. Future work will be needed to resolve predictions of these mechanisms.

### The functional role of the C-terminus of α-syn in oxidation and mitochondrial motility

In contrast to the N-terminus, the acidic and glutamate-rich C-terminus is unstructured [52] and has been implicated in modulating the membrane binding of α-syn [1, 43, 44]. aa120–140, which correspond to the glutamate-rich region, were found to weakly associate with lipids [53]. The C-terminus has been implicated in protecting α-syn from aggregation [54], with phosphorylation at Y125, Y133, and Y135 suppressing α-syn aggregation and toxicity [18, 55], and hyper-phosphorylation of α-syn affecting membrane-binding properties and subcellular distribution [56]. Therefore, while we postulate that the α-syn C-terminus has key roles during mitochondrial health perhaps due to its structural conformation, further investigations will be needed to identify how post-translational modifications (PTMs) within the C-terminus contribute to mitochondrial oxidation and motility.

Alternatively, since α-syn C-terminus has been implicated in multiple protein interactions [40, 41] with phosphorylation of S129 and Y125 influencing protein–protein interactions [57], perhaps aberrant interactions between the C-terminus and proteins in the mitochondrial quality control, turnover, and/or motor proteins could cause the mitochondrial oxidation and motility defects we observe. Indeed, PINK1 interacts with α-syn and evades α-syn-induced neurotoxicity by activating autophagy [58]. Consistent with this, we found that α-syn C-terminus was essential for α-syn-PINK1 associations (Fig. 6A–D, S10). Further, the rescue of α-syn-mediated mitochondrial damage and neuronal cell death we observe with excess PINK1 could result due to PINK1-mediated activation of pro-survival factors mTOR/AKT [59] and/or anti-apoptotic factors Bcl-2 [60]. Since PINK1 can also promote ubiquitination and proteasome degradation [61], perhaps degradation of excess α-syn could eliminate α-syn-mediated mitochondrial damage. Indeed, certain PTM modified forms of α-syn (soluble oligomers, dopamine-modification, and/or S129E) can bind translocator of the outer membrane 20 (TOM20), preventing its interaction with its co-receptor, TOM22, inhibiting mitochondrial protein import, impairing mitochondrial function, and elevating ROS production [62].

The α-syn C-terminus (either by itself or with other ‘helper” proteins) could associate with molecular motors for the motility of mitochondria. Indeed, α-syn and mitochondria are co-localized (Fig. S1A), α-syn moves bi-directionally within axons similar to mitochondria [21], and both α-syn and mitochondria can associate with kinesin and dynein [63, 64]. However, whether α-syn directly associates with milton and miro, the two proteins that link both kinesin-1 and dynein to mitochondria for motility [65, 66] is unknown. Alternatively, since PINK1 forms a multi-protein complex with milton and miro [67], α-syn-PINK1 associations could mediate mitochondrial trafficking. Therefore, since mitochondrial damage activates PINK1 accumulation and Parkin activation leading to ubiquitination and degradation of miro [68], perhaps α-syn C-terminus-mediated mitochondrial damage triggers the retrograde motility of oxidized mitochondria (Fig. 4) destined for mitophagy. Further investigations will be needed to test predictions of this proposal.

## Materials and methods

### Drosophila genetics

Transgenic *Drosophila* lines UAS-α-syn^WT^, UAS-α-syn^Δ71–82^, UAS-α-syn^1–120^, [17], UAS-α-syn^LP3^ [15], UAS-α-syn^WT^-eGFP [69], UAS-Drp1, Drp1^KG03815^/CyO, UAS-hMFN2, UAS-MitoTimer, UAS-mito-roGFP2-ORP1, UAS-mito-roGFP2-GRX1, UAS-HSPA (HSP70, BDSC), UAS-dPINK1/CyO [70], and UAS-Parkin [35] were used (Table S1). Neuronal drivers Appl-GAL4 (pan neuronal) and pGAL4-62B SG26-1 (8 motor neurons) were used for neuronal expression of transgenic lines [27, 28]. Genetics crossings were done as in [27, 28]. Unless otherwise stated, flies were reared at 29 °C, 60% humidity. In all cases non-tubby female 3rd instar larvae were selected. Sibling tubby larvae were evaluated as controls. Reciprocal crossings were also done to confirm observations.

### Larval brain immunohistochemistry and quantification of mitochondrial morphology

Third instar larval brains were isolated by brain pulls in which larval segmental nerves remained attached. Larval brains were then fixed (4% paraformaldehyde) and immunostained (cytochromeC 1:500, Abcam). Images of segmental nerves were collected using a Nikon Eclipse TE 2000U microscope at ×90 using the ×60 objective with 1.5× gain (Nikon, Melville, NY, USA). For each genotype, >250 mitochondria from at least six confocal optical images from the anterior, middle, and posterior regions of six larvae were imaged, and mitochondrial area [6] and axonal accumulations [24] were measured using NIH ImageJ.

### TUNEL assay

Brain pulls were performed on third instar larvae: brains were fixed (4% paraformaldehyde) and permeabilized (5% saponin) prior to incubation in TdT enzyme: fluorescein-dUTP solution (1:10) (TUNEL assay - In Situ Cell Death Detection Kit (Roche)). Brains were imaged (×40) using a Nikon Eclipse TE 2000U microscope. The number of cells positive for TUNEL was quantified using NIH ImageJ software from at least 5 larval brains per genotype.

### Analysis of mitochondria size and health using in vivo reporters MitoTimer and Mito-roGFP2

Larvae expressing MitoTimer, Mito-roGFP2-ORP1, or Mito-roGFP2-GRX1 were dissected and imaged under physiological conditions in dissection buffer as previously described [71]. MitoTimer-568nm/MitoTimer-488nm or Mito-roGFP2-488 nm/Mito-roGFP2-405 nm were simultaneously visualized within larval segmental nerves using a NikonTE-2000E inverted fluorescence microscope with a beam splitter containing narrow single-band GFP/DsRED or YFP/GFP filters, a Cool Snap HQ cooled CCD camera, and a ProScan II high speed shutter (100 mm/s) for simultaneous imaging as previously done [72, 73]. For each larva, four sets of movies at an imaging window frame size of 100 Microns at 150 frames were taken from the middle region of the larvae at an exposure of 500 ms. Refer to supplemental information for details regarding photostability/sensitivity of MitoTimer and Mito-roGFP2. Kymographs were generated using Metamorph software and 568/488 nm or 488/405 nm movies were split for analysis. From a total of 5 larvae, a set of 20 movies containing a cumulative total of >120 mitochondria were imaged for each environmental variable, pharmacological agent, and/or genotype. Mitochondrial areas were measures as described above using NIH ImageJ [6]. Relative 568/488 nm (colored: red/green) or 405/488 nm (colored: ImageJ LUT-Fire) intensity ratios were obtained for each individual mitochondrial trajectory from each movie. The directionality of mitochondria labeled with MitoTimer (568 or 488 nm) or Mito-roGFP2 (405 or 488 nm) was analyzed using a MATLAB-based custom particle tracer program as previously done [74]. Briefly, for each genotype, individual mitochondria were automatically classified as being either stationary, anterograde, retrograde, or reversing. The fraction of stationary mitochondria per animal is the average of the stationary cargo fraction calculated for each of the four, time-lapse movies. Reversing refers to a mitochondrion that has at least one switch event between anterograde and retrograde motility. Both anterograde or retrogradely moving mitochondria show a net movement in the respective direction without pausing or reversing [74].

### Larval feeding, stress conditions, immunohistochemistry, and in vivo imaging

For pharmacological feeding experiments, third instar larvae were grown in fly flood containing buffer (0.01% DMSO), 1 µM BFA1 (Cayman Chemical), 25 mM H_2_O_2_ (Fisher), 5 mM DA (Fisher), or 0.5 mM GSH (Fisher) (Table S1) dissolved in 0.01%DMSO for 3 h prior to dissection and whole-mounting of larval axons for in vivo simultaneous dual-view imaging of either MitoTimer (568/488 nm), Mito-roGFP2-ORP1 (405/488 nm) or Mito-roGFP2-GRX1 (405/488 nm) as described above. For Mdivi1 feeding, larvae were fed food containing 10 µM Mdivi-1 (Fisher) dissolved in 0.01% DMSO, or food containing 0.01% DMSO alone, for 8 h prior to dissection and subsequent in vivo simultaneous dual-view imaging of MitoTimer or immunohistochemistry with cytochrome C (Abcam, 1:500) as described above. Note that feeding larvae at high concentrations of Mdivi-1 (≥500 µM) induced lethality; feeding heterozygous DRP1 mutant larvae Mdivi-1 at 10 µM (8 h) caused elongated mitochondria (Fig. 5A). For stress conditions, larvae expressing MitoTimer were encapsulated in a tube containing dissection buffer and placed in either a bath of room temperature water (22 °C), cold water (4 °C), warm water (37 °C), adhered to a low speed vortex, or treated with a global bath of 500 nM BFA1 (Cayman Chemicals) for 1 h prior to dissection and in vivo imaging.

### JC-1 assay

Larvae were dissected and treated with JC-1 (1:800, [75]) for 10 min prior to imaging of larval segmental nerves using a NikonTE-2000E inverted fluorescence microscope with a beam splitter containing narrow single-band GFP/DsRED filters. Accumulation JC-1 dye at mitochondrial membranes depends on ΔΨm: JC-1 distributes as monomers (J-monomers) with low ΔΨm (excitation/emission: 485/535 nm—green) and distributes as aggregates (J-aggregates) with high ΔΨm (excitation/emission: 535/595 nm—red) [76]. Quantitative analysis of red/green fluorescence intensity ratio was performed using NIH ImageJ to measure mitochondrial ΔΨm in larval segmental nerves.

### Western blot and immunoprecipitation analysis

Adult fly brains were collected and homogenized in acetate buffer (10 mM HEPES, pH 7.4, 100 mM K acetate, 150 mM sucrose, 5 mM EGTA, 3 mM Mg acetate, 1 mM DTT, protease inhibitors (Roche), phosphatase inhibitor (Pierce)). Brain homogenates were centrifuged at 1000 × *g* for 15 min at 4 °C. The resulting supernatant (PNS) was then denatured (NuPage LDS), ran on 4–12% Bis-Tris gels (Invitrogen), and used for western blotting (α-syn (BD Biosciences 1:1000)), CytC (Abcam, 1:1000), dPINK1 ([70], 1:250), dParkin ([77], 1:250), DRP1 ([78], 1:250), or Tubulin (Abcam, 1:1000). Images from 3 to 5 blots were quantified using ImageLab and NIH ImageJ.

For isolation of heavy membranes, PNS from fly brain homogenates were further fractionated into soluble fractions (Sol), heavy membrane (P1), and vesicle fractions (VF) by sucrose gradient ultra-centrifugations as previously done [79, 80] using lysis buffer (4 mM HEPES, 320 mM sucrose pH 7.4) containing a phosphatase and protease inhibitor cocktail (Pierce). The VF, Sol, and P1 fraction were removed and used in western blot analysis. Heavier membranes and mitochondria were found in P1 and vesicles and membrane-associated proteins were found in the VF. Th extent of cytC release from cytosol to mitochondria was measured by immunoblotting P1 and Sol for CytC (Abcam, 1:1000). Quantification analysis across 3 independent experiments was performed (Imagelab software) to determine the Sol/P1 ratio of cytC, which was normalized to the Sol/P1 ratio of tubulin (Abcam, 1:1000) for each genotype.

For immunoprecipitation analysis, adult brains were homogenized in acetate buffer as previously described [79, 80]. The lysate was centrifuged at 1000 × *g* for 10 min at 4 °C and the resulting PNS was incubated overnight with α-syn antibody (BD Biosciences) at 4 °C. Protein A/G Magnetic Beads (Pierce) were then added, incubated at room temperature for 1 h, and eluted in low pH elution buffer (Pierce) as previously described [73]. The low pH was neutralized (Tris pH 8.8) and the concentration of the α-syn pull down was determined (Bicinchoninic acid (BCA) protein assay, Pierce). Western blot analysis was used to evaluate the extent and purity of the α-syn IP. In addition, dPINK1 ([70], 1:250), dParkin ([77], 1:250), or DRP1 [78] were evaluated in the α-syn pulldown as previously done [73].

### Statistical analysis

The statistical analysis used for each experiment is indicated in each figure legend. First power and sample size (*n*) calculations were performed on Minitab18 for each experimental paradigm: comparing 2 means from 2 samples, with two-sided equality to identify the sample size that corresponds to a power of 0.9 with *α* = 0.01. Analysis was conducted by multiple persons blinded. At least 5–6 larvae were used for each experiment. For western blot quantifications, *α* = 0.05; therefore, three independent experiments were performed. To select the appropriate statistical test, data distributions for each transport dynamic analyzed were first checked for normality using the nortest package of R: the Lilliefors test and Anderson–Darling test. Statistical significance of normal distributions was calculated by one-way ANOVA/post hoc analysis to reduce Type I error, followed by two-sample two-tailed Student’s *t* tests to test to compare individual groups in Excel and Minitab18. Statistical analysis reported in figures report results from Student’s *t* tests, as results from ANOVA/post hoc and Student’s *t*-tests were consistent. Data obtained from NIH ImageJ, MATLab, Image Lab, or Metamorph/Metavue were analyzed in Excel and Minitab18. Overlaid dot plots were constructed for all figures using OriginLab/OriginPro.

### Key resource table

**Table:** 

Resource	Source	Identifier
*Antibodies and dyes*		
Mouse anti-Tubulin (DM1A)	Abcam	Cat# ab7291 RRID: AB_2241126
Mouse anti-Cytochrome C (7H8.2C12)	Abcam	Cat# ab13575 RRID: AB_ 300470
Mouse anti-α-synuclein (clone 42)	BD Biosciences	Cat# 610787 RRID: AB_398108
Rabbit anti-dPINK1	Laboratory of Bingwei Lu	Yang et al. [70]
Rabbit anti-Parkin	Laboratory of Leo Pallanck	Greene et al. [77]
Rabbit anti-DRP1	Laboratory of Leo Pallanck	Poole et al. [78]
Anti-Mouse Alexa Fluor® 488	Thermofisher	Cat# A11001 RRID: AB_2534069
Anti-Mouse Alexa Fluor® 568	Thermofisher	Cat# A11004 RRID: AB_2534072
Anti-Rabbit Alexa Fluor® 488	Thermofisher	Cat# A11008 RRID: AB_143165
Anti-Rabbit Alexa Fluor® 568	Thermofisher	Cat# A11011 RRID: AB_143157
Anti-Mouse secondary antibody, HRP	Thermofisher	Cat# 32430 RRID: AB_1185566
Anti-Rabbit secondary antibody, HRP	Thermofisher	Cat# 32460 RRID: AB_1185567
JC-1 Mitochondrial Membrane Potential Assay Kit	Cayman Chemical	Cat# 10009172 CAS: 3520-43-2
In situ Cell Death Detection Kit, Fluorescein	Roche	Cat# 11684795910 Version# 17
*Chemicals, peptides, and recombinant proteins*		
Protease inhibitor cocktail	Pierce	Cat# PIA32965 RRID: N/A
Phosphatase Inhibitor	Pierce	Cat# PI88667 RRID: N/A
Protein A/G Magnetic Beads	Pierce	Cat# PI88802 RRID: N/A
Vecta Shield Mounting Medium	Fisher	Cat# NC9265087 RRID: N/A
Bafilomycin-A1 (BFA1)	Cayman Chemical	Cat# 11038 PubChem: 6436223
Hydrogen peroxide (H_2_O_2_)	Fisher Scientific	Cat# S25360 PubChem: 784
Diamide (DA) (N,N-Dimethylformamide)	Fisher Scientific	Cat# D119-500 PubChem: 6228
Glutathione reduced (GSH)	Fisher Scientific	Cat# BP252110 PubChem: 124886
Mitochondrial Division Inhibitor, Mdivi-1	Fisher Scientific	Cat# 47-585-610MG PubChem: 3825829
*Experimental models: D. melanogaster* *organisms/strains*		
P{Appl-GAL4.G1a}1, y^1^ w^*^	Bloomington Drosophila Stock Center	BDSC: 32040;FlyBase: FBst0032040
Appl-GAL4; T(2,3), CyO, TM6B, Tb^1^/Pin^88k^	Laboratory of Lawrence Goldstein	Gunawardena and Goldstein [27]
pGAL4-62B SG26-1	Bloomington Drosophila Stock Center	BDSC: 32257;FlyBase: FBst0032257
pGAL4-62B SG26-1; T(2,3), CyO, TM6B, Tb^1^/Pin^88k^	Laboratory of Lawrence Goldstein	Gunawardena [28]
UAS-α-synuclein^WT^ (UAS-α-syn^WT^)	Laboratory of Mel Feany	Periquet et al. [17]
UAS-α-synuclein^Δ71–82^ (UAS-α-syn^ΔNAC^)	Laboratory of Mel Feany	Periquet et al. [17]
UAS-α-synuclein^1–120^ (UAS-α-syn^1–120^)	Laboratory of Mel Feany	Periquet et al. [17]
UAS-α-synuclein^LP3^ (UAS-α-syn^LP3^)	Laboratory of Leo Pallanck	Trinh et al. [15]
w^1118^; P{UAS-MitoTimer}3	Bloomington Drosophila Stock Center	BDSC: 57323 FlyBase: FBst0057323
w^1118^; P{UAS-Mito-roGFP2-Orp1}10	Bloomington Drosophila Stock Center	BDSC: 67667 FlyBase: FBst0067667
w^1118^; P{UAS-Mito-roGFP2-Grx1}9	Bloomington Drosophila Stock Center	BDSC: 67664 FlyBase: FBst0067664
y^1^; P{SUPor-P} Drp1^KG03815^/CyO; ry^506^	Bloomington Drosophila Stock Center	BDSC: 13510 FlyBase: FBst0013510
UAS-dPINK1/CyO	Laboratory of Bingwei Lu	Yang et al. [70]
UAS-Parkin	Laboratories of Mel Feany and Leo Pallanck	Greene et al. [35]
w^1118^; UAS-α-synuclein^WT^-eGFP/TM6b (UAS-α-syn^WT^-eGFP/TM6B)	Laboratory of Pedro Domingos	Pocas et al. [69]
w^1118^; P{UAS-Hsap\HSPA1L.W}41.1	Bloomington Drosophila Stock Center	BDSC: 7454 FlyBase: FBst0007454
w^1118^; P{UAS-hMFN2.D}29/TM3, Sb^1^	Bloomington Drosophila Stock Center	BDSC: 59044 FlyBase: FBst0059044
w^*^; P{UAS-Drp1.D}3	Bloomington Drosophila Stock Center	BDSC: 51647 FlyBase: FBst0051647
*Software/algorithms*		
MATLAB-based particle tracking program	Laboratory of Danuser	Yang et al. [81]
ImageJ	Schneider et al. [82] https://imagej.net/	RRID: SCR_003070
Metamorph/Metavue Imaging Software	Molecular Devices, Sunnyvale, CA, USA	RRID: SCR_002368
Minitab18	https://www.minitab.com/en-us/	RRID: SCR_014483
Microsoft Excel	https://www.microsoft.com/en-gb/	RRID: SCR_016137
RStudio	http://www.rstudio.com/	RRID: SCR_000432
OriginLab/OriginPro	https://www.originlab.com/	RRID: SCR_014212

## Supplementary information

Supplemental materials

## Data Availability

The published article includes all datasets generated/analyzed for this study.

## Abbreviations

Drp1: dynamin-related protein 1
MFN: mitofusin
PD: Parkinson’s disease
α-syn: alpha synuclein
NAC: non-amyloid-β component
PINK1: PTEN-induced kinase 1
sPD: sporadic Parkinson’s disease
fPD: familial Parkinson’s disease
NMR: Nuclear magnetic resonance spectroscopy
MPTP: 1-methyl-4-phenyl-1,2,3,6-tetrahydropyridine
ER: endoplasmic reticulum
HSP70: heat shock protein 70
BFA1: bafilomycin-A1
H2O2: hydrogen peroxide
DA: diamide
GSH: glutathione reduced
ORP1: oxidant receptor peroxidase 1
GRX1: glutaredoxin 1
Mdivi-1: mitochondrial division inhibitor-1
Co-IP: co-immunoprecipitation
aa: amino acid
TUNEL: terminal deoxynucleotidyl transferase dUTP nick end labeling
OMM: outer mitochondrial membrane
IMM: inner mitochondrial membrane
PTM: post translational modification
Bcl-2: B-cell lymphoma 2
TOM20: translocator of the outer membrane 20
TOM22: translocator of the outer membrane 22
