# Supplementary material for: Differential mitochondrial roles for α-synuclein in DRP1-dependent fission and PINK1/Parkin-mediated oxidation
Source: Cell Death Dis. 2021 Aug 17;12(9):796. doi: 10.1038/s41419-021-04046-3 (PMC8371151; doi:10.1038/s41419-021-04046-3)
Supplement: Supplementary file 1 — Supplemental materials [file 41419_2021_4046_MOESM1_ESM.pdf]

Table S1: Fragmented mitochondria are not always oxidized. (NA=not analyzed)

**Condition or genotype**	**Mitochondrial morphology**	**Mitochondrial health**		
	Mitochondrial area (μm^2)	MitoTimer 568/488nm ratio (AU)	Mito-roGFP2-ORP1 405/488nm ratio (AU)	Mito-roGFP2-GRX1 405/488nm ratio (AU)
**WT (Buffer)**	---	---	---	---
**WT (BFA1)**	Elongated	more red (damaged)	more oxidation	more oxidation
**WT (H_2O_2)**	Fragmented	more red (damaged)	more oxidation	more oxidation
**WT (DA)**	Like WT	more red (damaged)	more oxidation	more oxidation
**WT (GSH)**	Like WT	Like WT	Like WT	Like WT
**WT (22°C)**	---	---	NA	NA
**WT (37°C)**	Fragmented	more green (healthy)	NA	NA
**WT (4°C)**	Elongated	more red (damaged)	NA	NA
**WT (Vortex)**	Fragmented	more red (damaged)	NA	NA
**Excess Drp1**	Fragmented	more red (damaged)	NA	NA
**Excess MFN2**	Elongated	more green	NA	NA

Table S2: The N-terminus of α-synuclein causes mitochondrial fragmentation in a Drp1-dependent manner while the C-terminus of α-syn is responsible for mitochondrial oxidation and for the retrograde motility bias.

**Genotype**	**Mitochondria morphology**	**Mitochondria health**	**Mitochondrial Motility Cargo Population (%)**			
	Mitochondria area (µm^2)	MitoTimer 568/488nm ratio (AU)	anterograde	retrograde	reversing	stationary
**WT**	---	---	---	---	---	---
**α-syn^{WT}**	Fragmented	more red (damaged)	↓	↑	↑	↓
**α-syn$^{\Delta NAC}$**	Fragmented	Like WT & α-syn^{WT}	↓	↑	Like WT	Like WT
**α-syn$^{1\text{-}120}$**	Fragmented	Like WT	Like WT	Like WT	Like WT	Like WT
**WT (Mdivi1)**	Like WT	Like WT	Like WT	Like WT	Like WT	Like WT
**Drp1$^{+/-}$ (Mdivi1)**	Elongated	more red (damaged)	NA	NA	NA	NA
**α-syn^{WT} (Buffer)**	Fragmented	more red (damaged)	↓	↑	↑	↓
**α-syn^{WT} (Mdivi1)**	Like WT	Like WT	Like WT	Like WT	↑	Like WT
**α-syn$^{1\text{-}120}$ (Buffer)**	Fragmented	more green (healthy)	Like WT	Like WT	Like WT	Like WT
**α-syn$^{1\text{-}120}$ (Mdivi1)**	Like WT	more green (healthy)	Like WT	Like WT	Like WT	Like WT

Table S3: The C-terminus of α-syn is responsible for mitochondrial oxidation/depolarization via a PINK1/Parkin mechanism. ((NA=not analyzed)

**Genotype**	**Mitochondria morphology**	**Mitochondria health**	**Mitochondrial Motility Cargo Population (%)**			
	Mitochondria area (µm^2)	JC-1 568/488nm ratio (AU)	anterograde	retrograde	reversing	stationary
**WT (JC1)**	Like WT	Like WT	NA	NA	NA	NA
**α-syn^{WT} (JC1)**	fragmented	more green (depolarized)	NA	NA	NA	NA
**α-syn$^{1\text{-}120}$ (JC1)**	fragmented	Less green (↓ depolarized)	NA	NA	NA	NA
**α-syn^{WT};PINK1 (JC1)**	Like WT	Like WT	NA	NA	NA	NA
**α-syn^{WT};PARKIN (JC1)**	Like WT	Like WT	---	---	---	---

Figure S1

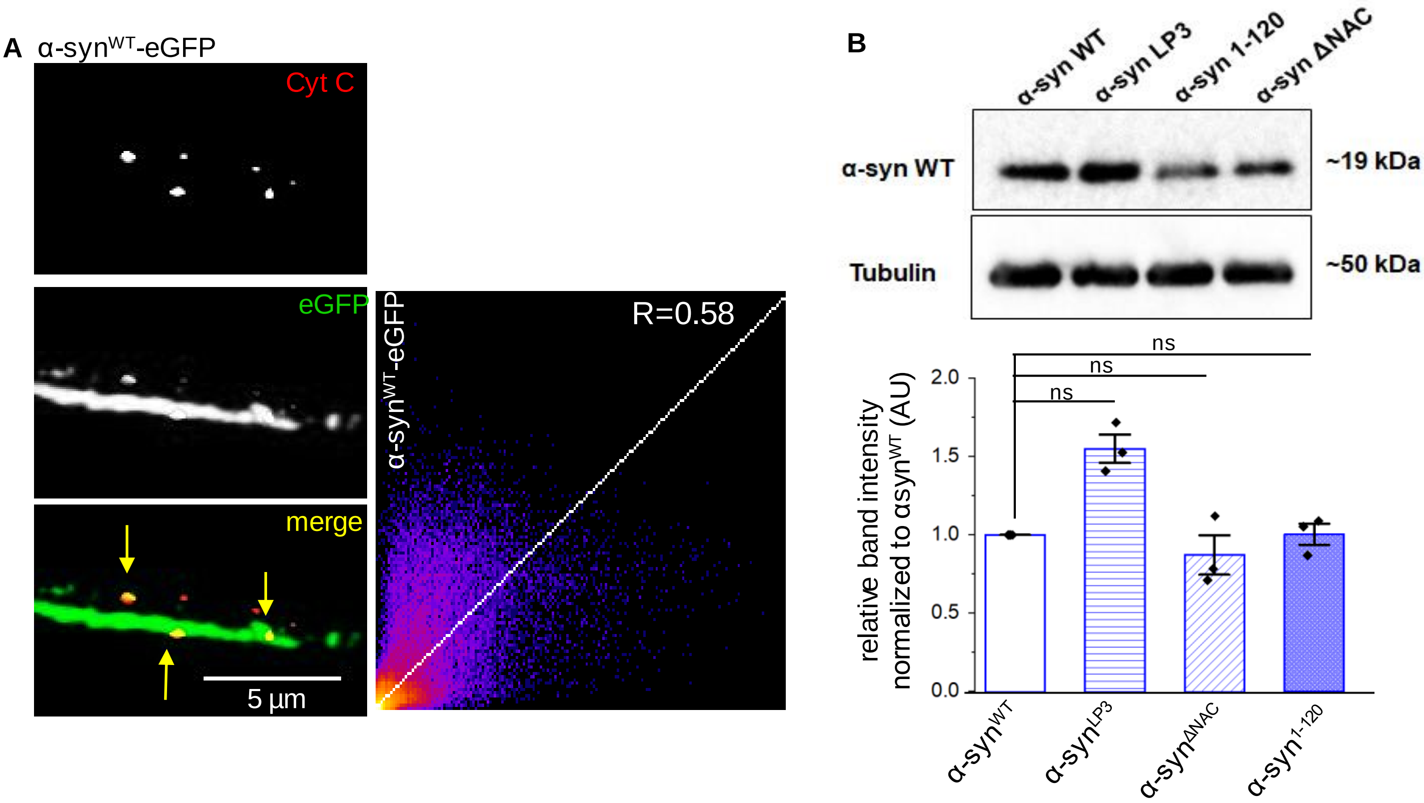
A
α-syn^WT-eGFP
Cyt C
eGFP
merge
5 µm
α-syn^WT-eGFP
R=0.58
B
α-syn WT
α-syn LP3
α-syn 1-120
α-syn ΔNAC
α-syn WT
Tubulin
~19 kDa
~50 kDa
ns
ns
ns
relative band intensity normalized to αsyn^WT (AU)
2.0
1.5
1.0
0.5
0.0
α-syn^WT
α-syn^LP3
α-syn^ΔNAC
α-syn^1-120

Figure S2

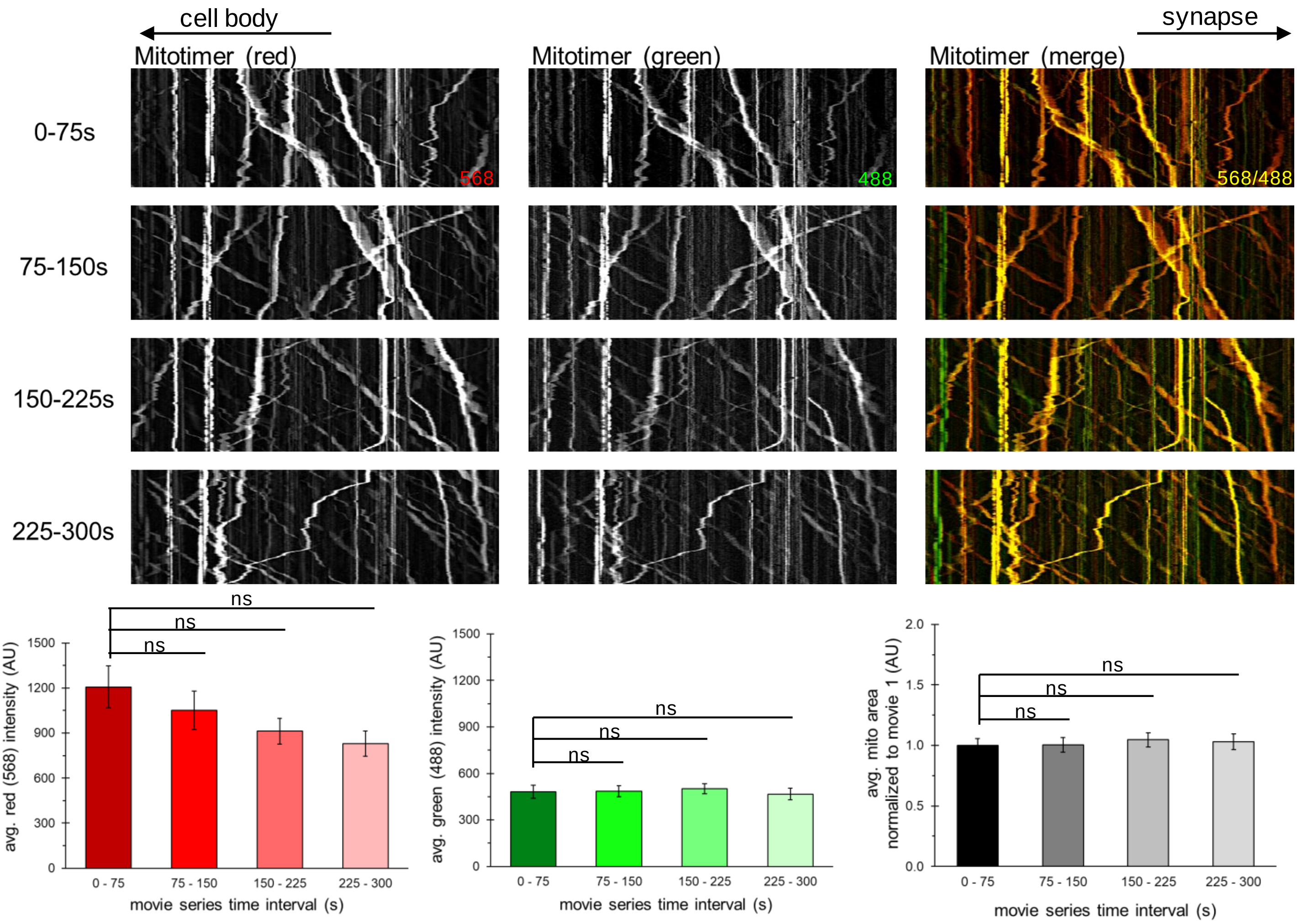
cell body
synapse
Mitotimer (red)
Mitotimer (green)
Mitotimer (merge)
0-75s
75-150s
150-225s
225-300s
568
488
568/488
ns
avg. red (568) intensity (AU)
avg. green (488) intensity (AU)
avg. mito area normalized to movie 1 (AU)
0 - 75
75 - 150
150 - 225
225 - 300
movie series time interval (s)

Figure S3

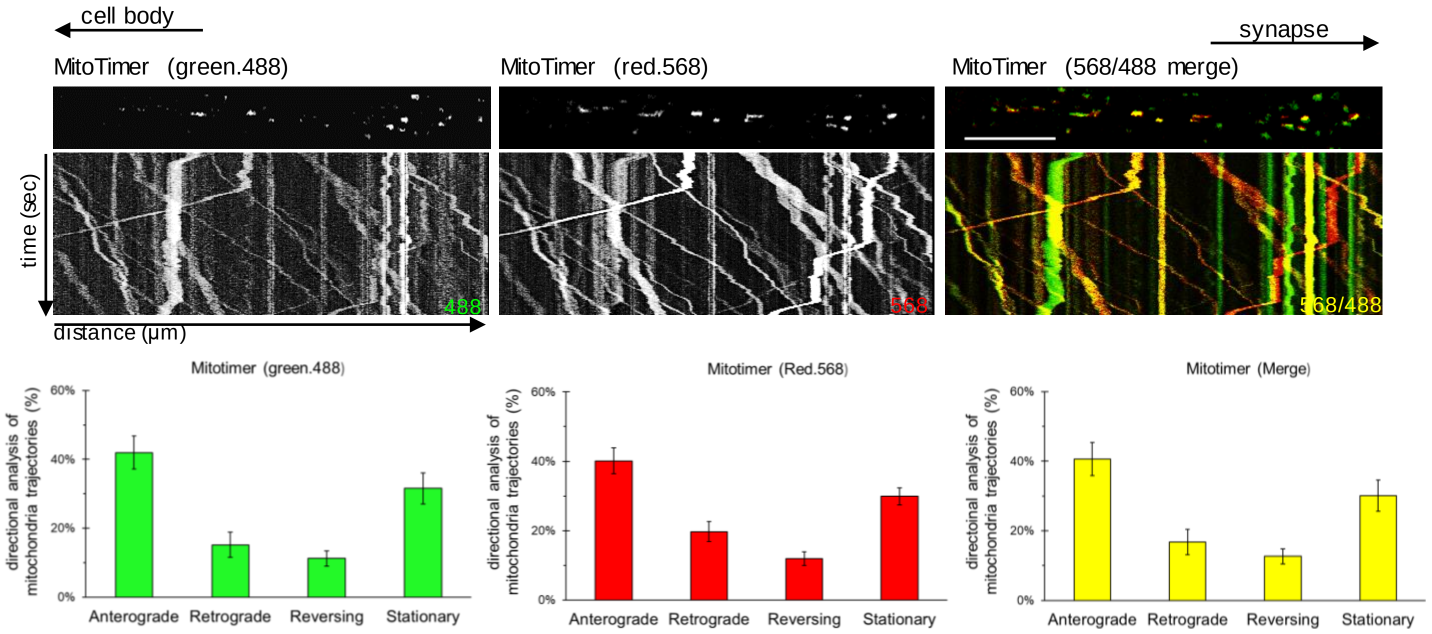

cell body
synapse
MitoTimer (green.488)
MitoTimer (red.568)
MitoTimer (568/488 merge)
time (sec)
distance (μm)
488
568
568/488
Mitotimer (green.488)
Mitotimer (Red.568)
Mitotimer (Merge)
directional analysis of mitochondria trajectories (%)
directoinal analysis of mitochondria trajectories (%)
60%
40%
20%
0%
Anterograde
Retrograde
Reversing
Stationary

Figure S4

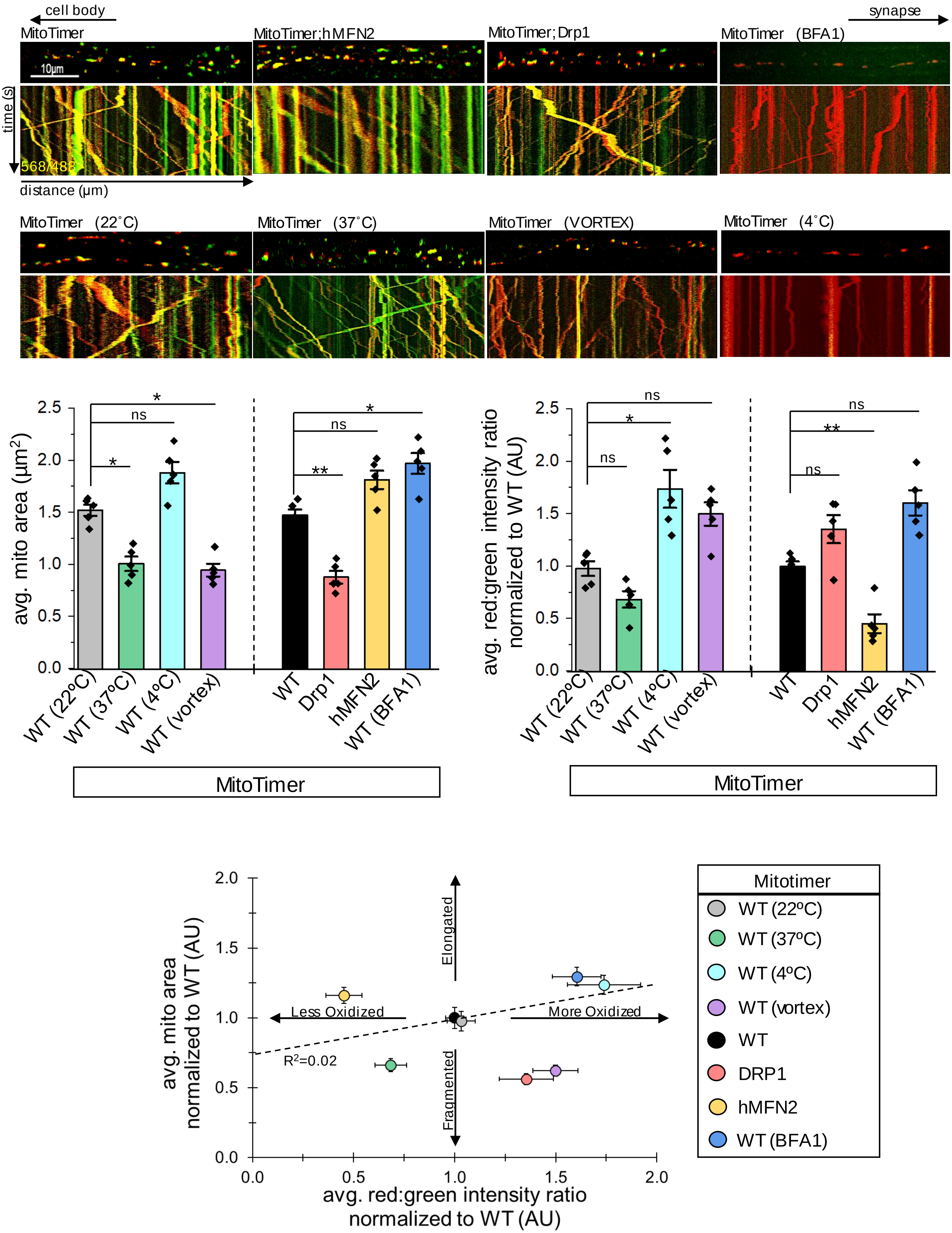

cell body
synapse
MitoTimer
MitoTimer;hMFN2
MitoTimer;Drp1
MitoTimer (BFA1)
10µm
time (s)
568/488
distance (µm)
MitoTimer (22°C)
MitoTimer (37°C)
MitoTimer (VORTEX)
MitoTimer (4°C)
avg. mito area (µm²)
WT (22ºC)
WT (37ºC)
WT (4ºC)
WT (vortex)
WT
Drp1
hMFN2
WT (BFA1)
MitoTimer
avg. red:green intensity ratio normalized to WT (AU)
MitoTimer
avg. mito area normalized to WT (AU)
avg. red:green intensity ratio normalized to WT (AU)
Elongated
Fragmented
Less Oxidized
More Oxidized
R²=0.02
Mitotimer
WT (22ºC)
WT (37ºC)
WT (4ºC)
WT (vortex)
WT
DRP1
hMFN2
WT (BFA1)

Figure S5

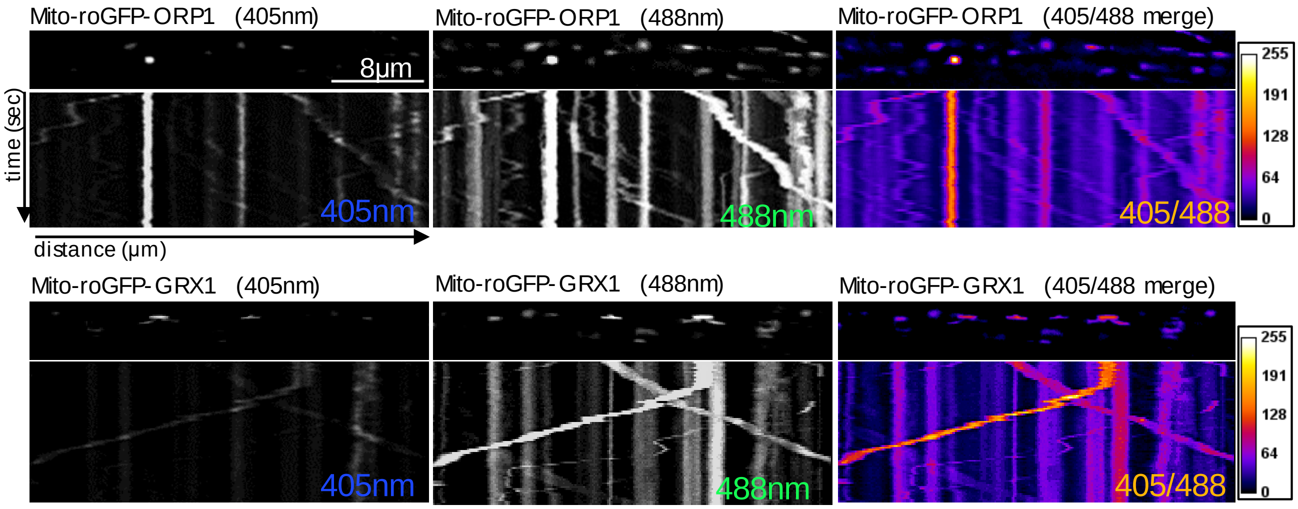

Mito-roGFP-ORP1 (405nm)
Mito-roGFP-ORP1 (488nm)
Mito-roGFP-ORP1 (405/488 merge)
8μm
time (sec)
distance (μm)
405nm
488nm
405/488
255
191
128
64
0
Mito-roGFP-GRX1 (405nm)
Mito-roGFP-GRX1 (488nm)
Mito-roGFP-GRX1 (405/488 merge)
405nm
488nm
405/488
255
191
128
64
0

Figure S6

A

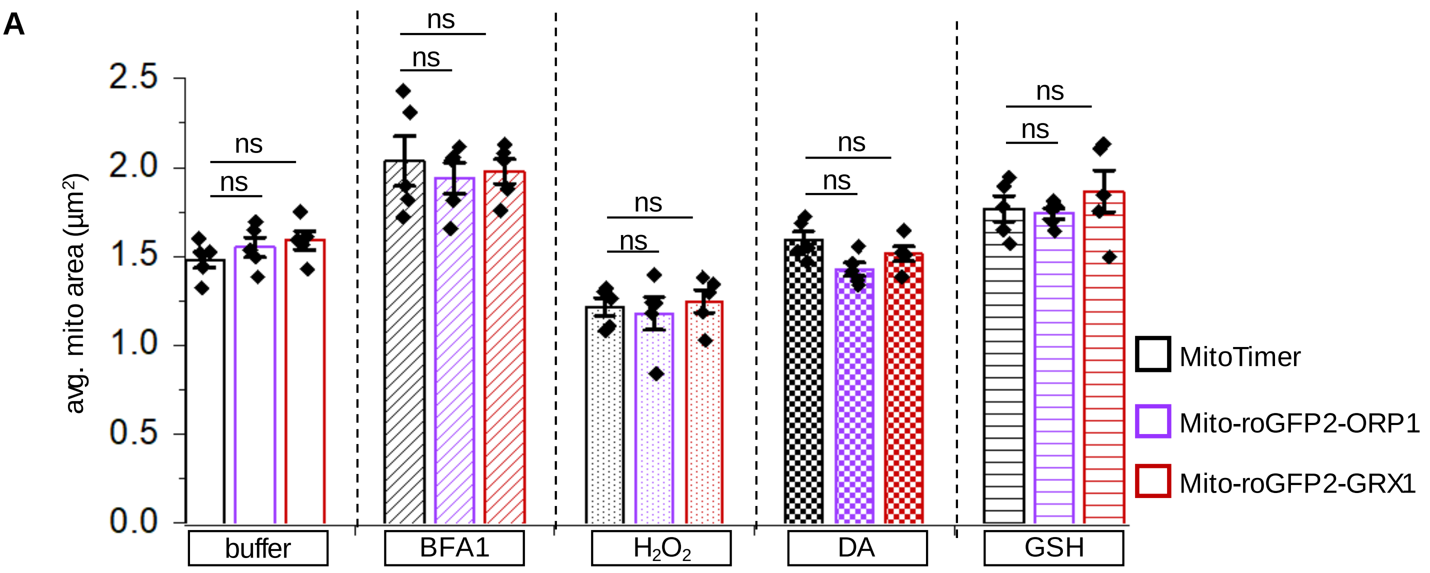

avg. mito area (μm²)
2.5
2.0
1.5
1.0
0.5
0.0
ns
buffer
BFA1
H₂O₂
DA
GSH
MitoTimer
Mito-roGFP2-ORP1
Mito-roGFP2-GRX1

B

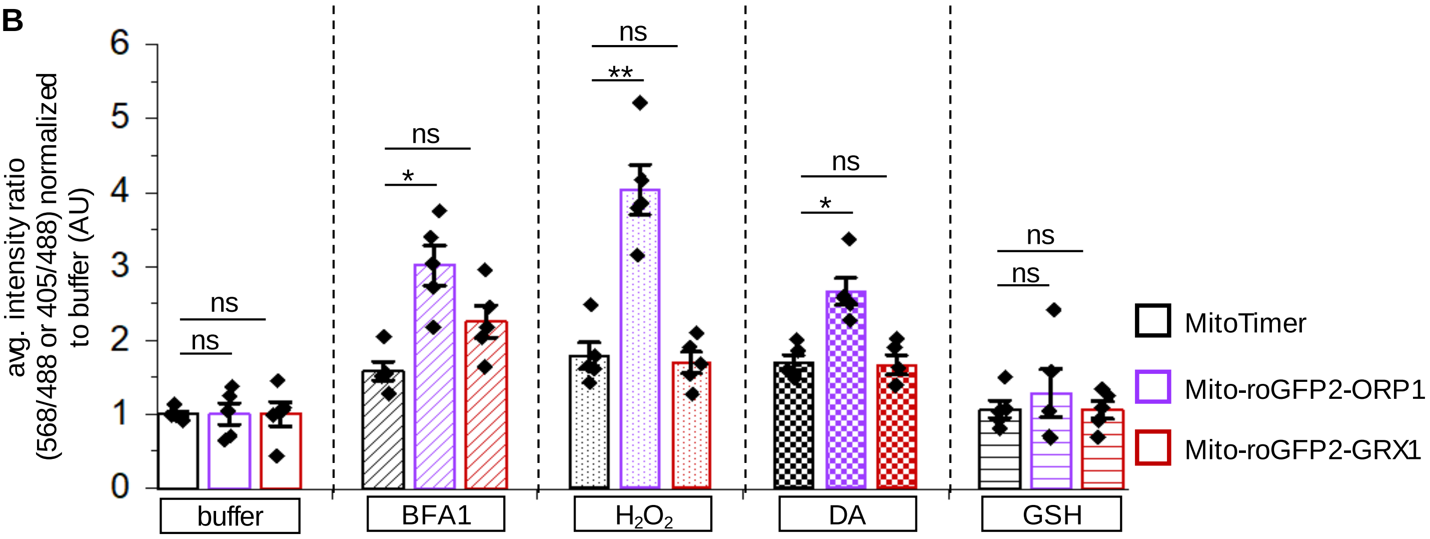

avg. intensity ratio (568/488 or 405/488) normalized to buffer (AU)
6
5
4
3
2
1
0
ns
*
**
buffer
BFA1
H₂O₂
DA
GSH
MitoTimer
Mito-roGFP2-ORP1
Mito-roGFP2-GRX1

Figure S7

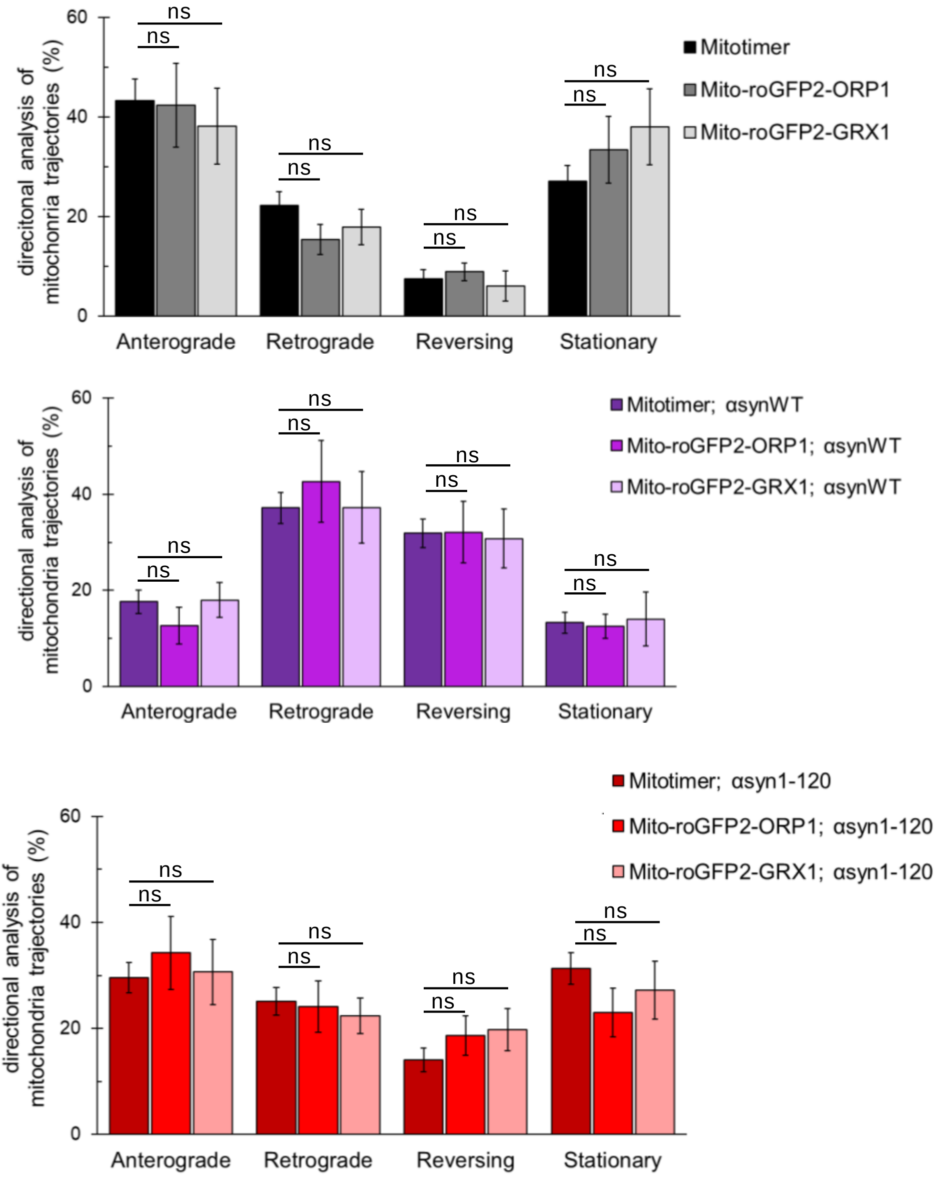

Mitotimer
Mito-roGFP2-ORP1
Mito-roGFP2-GRX1
direcitonal analysis of mitochonria trajectories (%)
60
40
20
0
ns
Anterograde
Retrograde
Reversing
Stationary
Mitotimer; αsynWT
Mito-roGFP2-ORP1; αsynWT
Mito-roGFP2-GRX1; αsynWT
directional analysis of mitochondria trajectories (%)
Mitotimer; αsyn1-120
Mito-roGFP2-ORP1; αsyn1-120
Mito-roGFP2-GRX1; αsyn1-120
directional analysis of mitochondria trajectories (%)

Figure S8

A

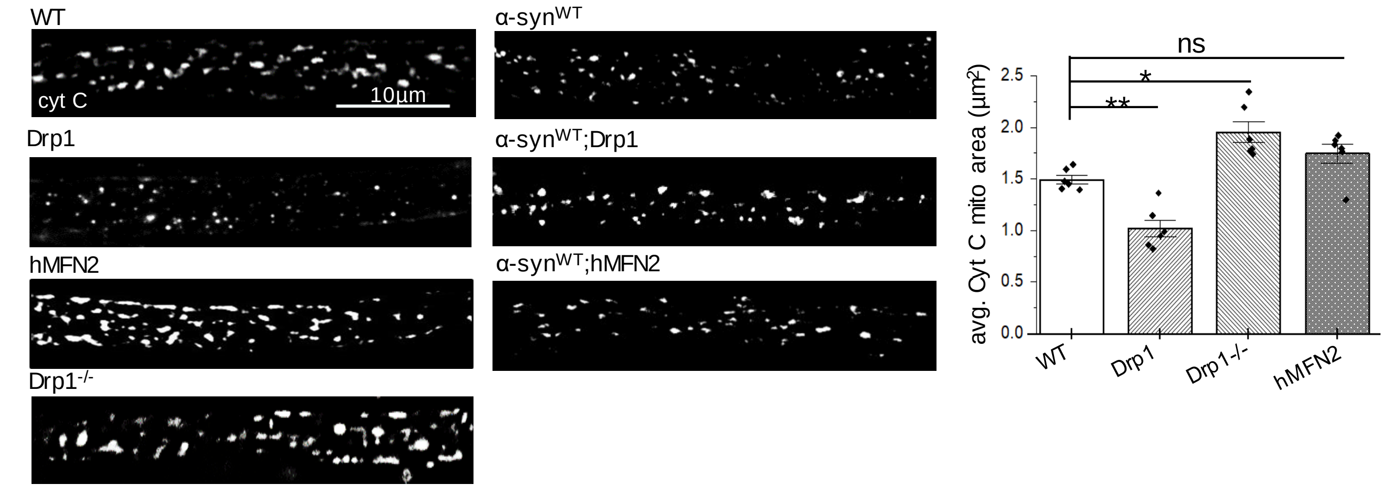
WT
cyt C
10µm
α-syn^WT
Drp1
α-syn^WT;Drp1
hMFN2
α-syn^WT;hMFN2
Drp1^-/-
ns
*
**
avg. Cyt C mito area (µm²)
2.5
2.0
1.5
1.0
0.5
0.0
WT
Drp1
Drp1-/-
hMFN2

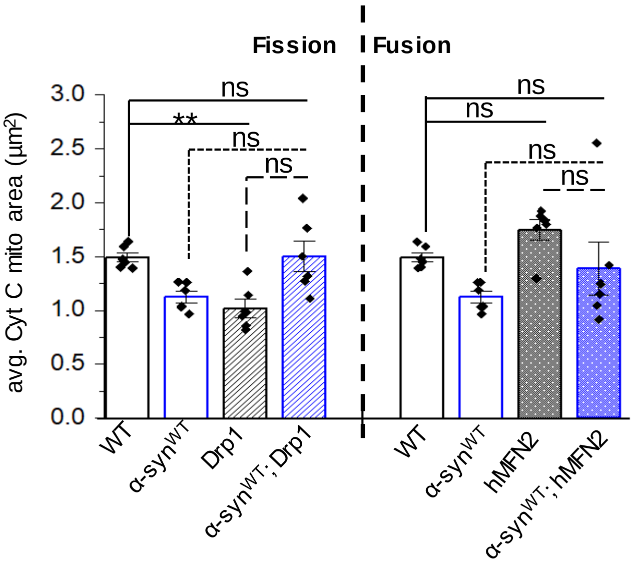
Fission
Fusion
3.0
2.5
2.0
1.5
1.0
0.5
0.0
ns
**
ns
ns
ns
ns
ns
ns
avg. Cyt C mito area (µm²)
WT
α-syn^WT
Drp1
α-syn^WT; Drp1
WT
α-syn^WT
hMFN2
α-syn^WT; hMFN2

B

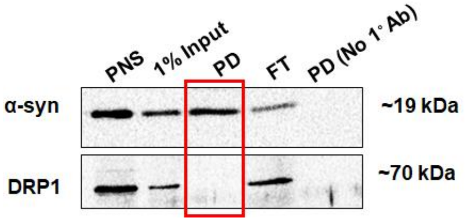
PNS
1% Input
PD
FT
PD (No 1° Ab)
α-syn
DRP1
~19 kDa
~70 kDa

Figure S9

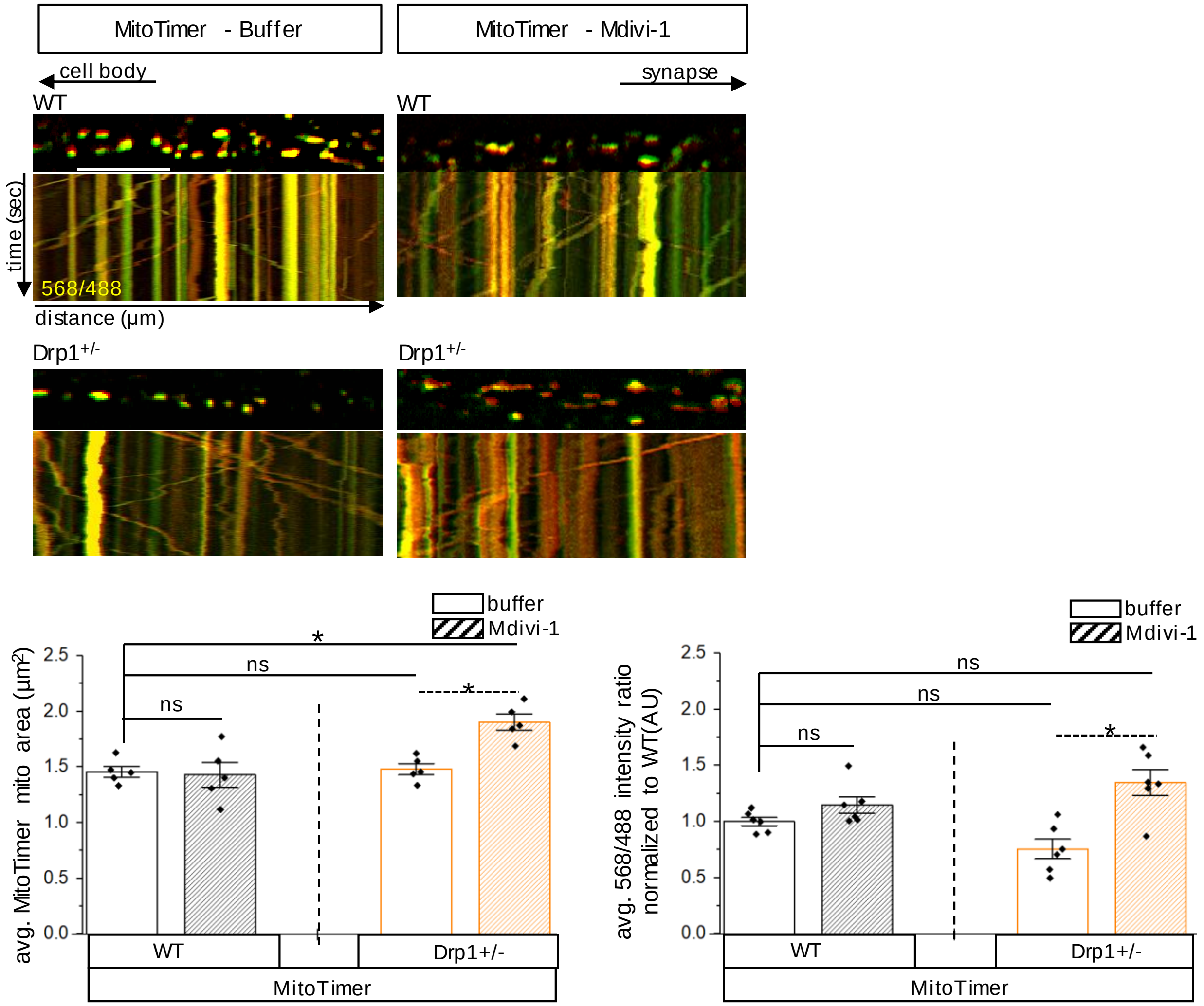

MitoTimer - Buffer
MitoTimer - Mdivi-1
cell body
synapse
WT
WT
time (sec)
568/488
distance (μm)
Drp1+/-
Drp1+/-
buffer
Mdivi-1
*
ns
ns
*
avg. MitoTimer mito area (μm²)
2.5
2.0
1.5
1.0
0.5
0.0
WT
Drp1+/-
MitoTimer
buffer
Mdivi-1
ns
ns
ns
*
avg. 568/488 intensity ratio normalized to WT(AU)
2.5
2.0
1.5
1.0
0.5
0.0
WT
Drp1+/-
MitoTimer

Figure S10

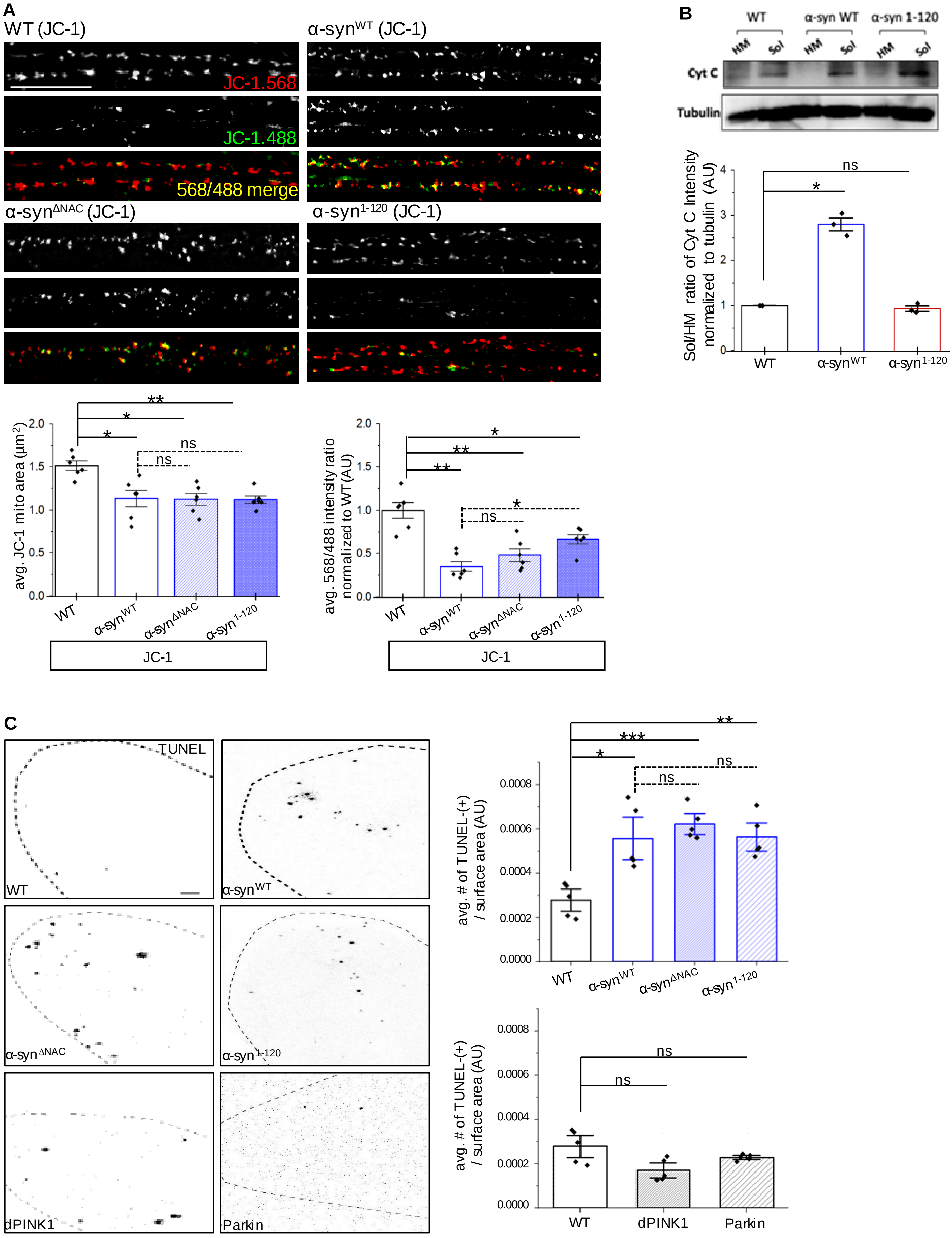
A
WT (JC-1)
α-synWT (JC-1)
JC-1.568
JC-1.488
568/488 merge
α-synΔNAC (JC-1)
α-syn1-120 (JC-1)
avg. JC-1 mito area (μm2)
2.0
1.5
1.0
0.5
0.0
**
*
*
ns
ns
WT
α-synWT
α-synΔNAC
α-syn1-120
JC-1
avg. 568/488 intensity ratio normalized to WT (AU)
2.0
1.5
1.0
0.5
0.0
*
**
**
*
ns
WT
α-synWT
α-synΔNAC
α-syn1-120
JC-1
B
WT
α-syn WT
α-syn 1-120
HM
Sol
HM
Sol
HM
Sol
Cyt C
Tubulin
Sol/HM ratio of Cyt C Intensity normalized to tubulin (AU)
4
3
2
1
0
ns
*
WT
α-synWT
α-syn1-120
C
TUNEL
WT
α-synWT
α-synΔNAC
α-syn1-120
dPINK1
Parkin
avg. # of TUNEL-(+) / surface area (AU)
0.0008
0.0006
0.0004
0.0002
0.0000
**
***
*
ns
ns
WT
α-synWT
α-synΔNAC
α-syn1-120
avg. # of TUNEL-(+) / surface area (AU)
0.0008
0.0006
0.0004
0.0002
0.0000
ns
ns
WT
dPINK1
Parkin

Figure S11

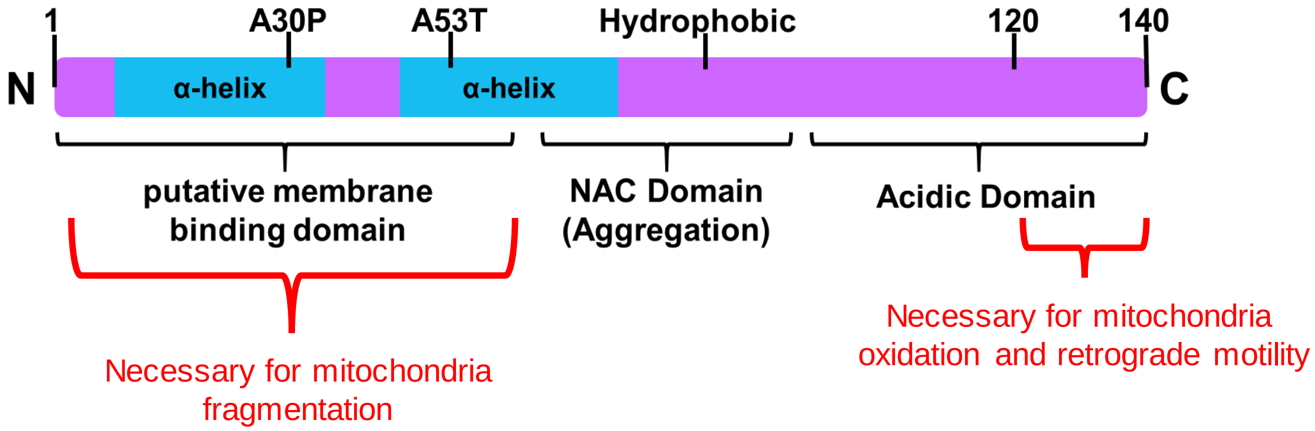
1
A30P
A53T
Hydrophobic
120
140
N
α-helix
α-helix
C
putative membrane binding domain
NAC Domain (Aggregation)
Acidic Domain
Necessary for mitochondria oxidation and retrograde motility
Necessary for mitochondria fragmentation
